# Deficiency of exchange protein directly activated by cAMP (EPAC)-1 in mice augments glucose intolerance, inflammation, and gut dysbiosis associated with Western diet

**DOI:** 10.1186/s40168-022-01366-0

**Published:** 2022-11-04

**Authors:** Preeti Dinesh Virwani, Lin Cai, Patrick Ka Kit Yeung, Gordon Qian, Yingxian Chen, Lei Zhou, Jason Wing Hon Wong, Yu Wang, Joshua Wing Kei Ho, Kui Kai Lau, Pei-Yuan Qian, Sookja Kim Chung

**Affiliations:** 1 Faculty of Medicine; Faculty of Innovation Engineering, Macau University of Science and Technology, Macau Special Administrative Region (S.A.R.), China; 2 School of Biomedical Sciences, Li Ka Shing (LKS) Faculty of Medicine, The University of Hong Kong, Hong Kong S.A.R., China; 3 Department of Medicine, LKS Faculty of Medicine, The University of Hong Kong, Hong Kong S.A.R., China; 4 Department of Ocean Science and Division of Life Science, Hong Kong University of Science and Technology, Kowloon, Hong Kong S.A.R. China; 5 Laboratory of Data Discovery for Health Limited (D24H), Hong Kong Science Park, Hong Kong S.A.R., China; 6 Department of Pharmacology and Pharmacy, LKS Faculty of Medicine, The University of Hong Kong, Hong Kong S.A.R., China; 7 State Key Laboratory of Pharmaceutical Biotechnology, The University of Hong Kong, Hong Kong S.A.R., China; 8 State Key Laboratory of Brain and Cognitive Sciences, The University of Hong Kong, Hong Kong S.A.R., China; 9 Southern Marine Science and Engineering Guangdong Laboratory (Guangzhou), Guangzhou, 511458 China; 10 Dr. Neher’s Biophysics Laboratory for Innovative Drug Discovery, Macau University of Science and Technology, Macau S.A.R., China

**Keywords:** Epac, Gut microbiota, Western diet, Obesity, Inflammation, Glucose intolerance, Gut dysbiosis

## Abstract

**Background:**

Gut microbiota (GM) dysregulation, known as dysbiosis, has been proposed as a crucial driver of obesity associated with “Western” diet (WD) consumption. Gut dysbiosis is associated with increased gut permeability, inflammation, and insulin resistance. However, host metabolic pathways implicated in the pathophysiology of gut dysbiosis are still elusive. Exchange protein directly activated by cAMP (Epac) plays a critical role in cell-cell junction formation and insulin secretion. Here, we used homozygous Epac1-knockout (Epac1^–/–^), Epac2-knockout (Epac2^–/–^), and wild-type (WT) mice to investigate the role of Epac proteins in mediating gut dysbiosis, gut permeability, and inflammation after WD feeding.

**Results:**

The 16S rRNA gene sequencing of fecal DNA showed that the baseline GM of Epac2^–/–^, but not Epac1^–/–^, mice was represented by a significantly higher *Firmicutes* to *Bacteroidetes* ratio and significant alterations in several taxa compared to WT mice, suggesting that Epac2^–/–^ mice had gut dysbiosis under physiological conditions. However, an 8-week WD led to a similar gut microbiome imbalance in mice regardless of genotype. While Epac1 deficiency modestly exacerbated the WD-induced GM dysbiosis, the WD-fed Epac2^–/–^ mice had a more significant increase in gut permeability than corresponding WT mice. After WD feeding, Epac1^–/–^, but not Epac2^–/–^, mice had significantly higher mRNA levels of tumor necrosis factor-alpha (TNF-α) and F4/80 in the epididymal white adipose tissue (EWAT), increased circulating lipocalin-2 protein and more severe glucose intolerance, suggesting greater inflammation and insulin resistance in WD-fed Epac1^–/–^ mice than corresponding WT mice. Consistently, Epac1 protein expression was significantly reduced in the EWAT of WD-fed WT and Epac2^–/–^ mice.

**Conclusion:**

Despite significantly dysregulated baseline GM and a more pronounced increase in gut permeability upon WD feeding, WD-fed Epac2^–/–^ mice did not exhibit more severe inflammation and glucose intolerance than corresponding WT mice. These findings suggest that the role of gut dysbiosis in mediating WD-associated obesity may be context-dependent. On the contrary, we demonstrate that deficiency of host signaling protein, Epac1, drives inflammation and glucose intolerance which are the hallmarks of WD-induced obesity.

Video abstract

**Supplementary Information:**

The online version contains supplementary material available at 10.1186/s40168-022-01366-0.

## Background

A diet rich in high-glycemic carbohydrates and low fiber content, popularly known as the Western diet (WD), is evidently a significant contributor to the obesity and metabolic syndrome pandemic [1]. This has been responsible for substantial economic burden [2], morbidity, and mortality worldwide [3]. Over the past two decades or so, GM has been established as a critical driver of inflammation and insulin resistance associated with obesity [4–8]. The GM comprises innumerable microbes that mutually reside in the host’s gastrointestinal tract; the host benefits from an enormous pool of functional microbial components, which supposedly act as a remote endocrine “organ” and modulate the biochemical and physiological processes of the host [9, 10]. The GM is a dynamic entity with constantly changing microbial composition and functions due to various host and external factors, including age, diseases, antibiotics, and diet [11]. Imbalances in the GM have indeed been associated with several metabolic disorders, including obesity [4–8], diabetes [12], and nonalcoholic steatohepatitis [13]. More than 90% of the GM inhabiting the distal gut is represented by the bacterial phyla, *Firmicutes* and *Bacteroidetes* [14]. An increased *Firmicutes* to *Bacteroidetes* (f/b) ratio has been associated with the phenotype of obesity in humans and animal models [15]. Studies with gnotobiotic animals have shown that obesity can be transmitted through fecal transplantation alone [15, 16], suggesting that dysregulated GM can instigate adipogenesis. These data point to an integral relationship between the GM and host adipogenesis pathways and warrant investigation into host cellular pathways implicated in metabolic complications associated with GM dysbiosis.

The second messenger, cyclic adenosine monophosphate (cAMP), and its downstream effector, protein kinase A (PKA), signaling is well-known to mediate glucose homeostasis, insulin secretion, adiposity, and energy metabolism [17]. Epac proteins, which have the two main isoforms, Epac1 and Epac2A (Epac2), are PKA-independent downstream effectors of cAMP with guanine nucleotide exchange factor (GEF) activity for Ras-like small GTPases, Rap1, and Rap2. While Epac1 is ubiquitously expressed, Epac2 expression is confined to nervous tissue, beta cells [18, 19], and heart [20]. In addition, splice variants of Epac2 have been reported in adrenal glands (Epac2B) and liver (Epac2c) [21]. Several in vitro studies using Epac analogs have reported that Epac is involved in a myriad of cellular activities, including proliferation, secretion (vesicle trafficking, exocytosis), actin dynamics, cell-cell junction formation, and inflammation [22–24]. Previously, it was shown that Epac2 mediates insulin secretion, and high-fat diet (HFD)-fed Epac2^–/–^ mice exhibited significantly reduced insulin secretion and impaired glucose tolerance than WT mice during an intraperitoneal glucose tolerance test (IPGTT) [25]. On the other hand, we have reported that Epac1^–/–^ mice have lower plasma insulin levels in an IPGTT under physiological conditions, defects in insulin secretion, and lower glucose transporter 2 expression in the pancreas [26]. Additionally, HFD-fed Epac1^–/–^ mice had higher blood glucose levels compared to corresponding WT mice in an oral glucose tolerance test (OGTT) and insulin tolerance test (ITT) [26]*.* Taken together, these data show that both Epac1 and Epac2 are involved in insulin secretion, and defects in Epac signaling contribute to HFD-induced insulin resistance.

Furthermore, Epac1^–/–^ mice exhibited metabolic defects, including higher cholesterol, triglycerides, and body weight (BW), suggesting a mild metabolic syndrome under physiological conditions [26]. Moreover, Epac1^–/–^ mice developed more severe obesity and glucose intolerance upon HFD feeding [26]. Several metabolic and cellular defects, including gut permeability impairment, inflammation, and insulin resistance, are implicated in the pathogenesis of GM dysbiosis associated with obesity [27]. The disruption of the gut epithelial barrier precedes HFD-induced obesity in that compromised gut barrier allows higher uptake of bacterial endotoxins, which results in low-grade systemic inflammation (endotoxemia), eventually leading to insulin resistance [5, 27]. It has been reported that Epac1 regulates endothelial cell-cell junctional permeability through cadherin-mediated cell-cell adhesions and cytoskeletal reorganization [28, 29], and Epac1-deficient mice showed endothelial barrier dysfunction [30]. In addition, downstream of Epac, Rap-1 improved the endothelial cell permeability through adherens junctions and TJ proteins, ZO-1, and occludin [31].

Here, we hypothesized that Epac proteins mediate the pathological processes of gut dysbiosis associated with WD consumption and investigated the role of Epac proteins in gut permeability, inflammation and gut dysbiosis upon WD feeding using Epac1^–/–^ and Epac2^–/–^ mice.

## Materials and methods

### Animal studies

#### Animals

All animal studies were carried out according to the guidelines specified by the Committee on the Use of Live Animals in Teaching and Research (CULATR) at the University of Hong Kong (HKU). The present study was performed using Epac1 knockout (Epac1^–/–^) and Epac2A knockout (Epac2^–/–^) mice. Wild-type C57 (WT) mice served as the control group. Epac1^–/–^ mice were generated by performing targeted deletion of Epac1 using homologous recombination as previously described in detail [26]. Epac2^–/–^ mice were procured from Prof. Susumu Seino’s group [32]. Epac1^–/–^ and Epac2^–/–^ mice were backcrossed to C57BL/6N for 10 generations to generate mice of N10 background. The mice were housed at the HKU laboratory animal unit (LAU) under 12-h light and dark cycle with ad libitum access to a regular diet (RD) [Lab Diet 5001, Purina Milling Incorporated (PMI) Feeds, USA]. Genotyping was performed before each experiment to confirm the genotype of the mice as described previously for Epac1^–/–^ and Epac1 WT (Epac1^+/+^) gene [26], and Epac2^–/–^ and Epac2 WT (Epac2^+/+^) gene [32].

#### Western diet (WD) feeding

Eight-week-old male WT, Epac1^–/–^, and Epac2^–/–^ mice were provided ad libitum access to the WD (43 kcal % carbohydrate, 40 kcal % fat, 17 kcal % protein and cholesterol 1.5g/kg; D12079B, Research Diets Inc., USA) or RD (58, 28.5, and 13.5% of total calories from carbohydrates, protein and fat, respectively) for 8 weeks. Body weight (BW) was recorded weekly using a weighing scale (A&D GF-2000, A&D Company, Japan). The blood glucose was measured bi-weekly using a glucose meter (Contour Plus, Bayer HealthCare LLC, USA).

#### Collection of blood and tissue samples

The tissues for RNA, protein, and biochemical analysis were collected from 16-week-old mice (8-week-old + 8 weeks of RD or WD feeding). The 6-h-fasted mice were anesthetized with 125 mg/kg body weight ketamine and 7.5mg/kg body weight xylazine and sacrificed by cervical dislocation. The tissues were immediately fixed or snap-frozen in liquid nitrogen and stored at −80 °C until further processing for protein or RNA extraction.

#### Oral glucose tolerance test (OGTT)

OGTT was performed after the mice were fed either RD or WD for 7 weeks. A 20% of glucose solution was freshly prepared in filter-sterilized saline (0.9% of NaCl). The mice were fasted for 6 h, and glucose (1 g/kg body weight) was administered by oral gavage [5]. Blood glucose levels were determined using a glucose meter at 0, 15, 30, 45, and 90 min after oral glucose administration.

#### In vivo gut permeability assay

Fluorescein-isothiocyanate (FITC)-dextran (4000 Da) (Sigma-Aldrich, USA) was used as a tracer to measure the paracellular gut permeability as described previously [5]. Sixteen-week-old Epac1^–/–^, Epac2^–/–^, and WT mice (8-week-old + 8 weeks of RD or WD feeding) were fasted for 6 h, followed by administration of 4000-Da FITC-dextran (600 mg/kg body weight, 125 mg/ml stock solution) by oral gavage. The mice received an intraperitoneal (IP) injection of anesthetic, 125 mg/kg body weight ketamine, and 7.5 mg/kg body weight xylazine 55 min after the oral administration of FITC-dextran and the blood was collected by cardiac puncture 60 min after the FITC-dextran administration. Fifty microliters of serum samples was diluted with an equal volume of PBS (pH 7.4) and applied to the wells of an opaque 96-well plate. The FITC-dextran fluorescence intensity was recorded to determine the intestinal permeability [33] using a spectrophotometer (Spectra Max 340; Molecular Devices, USA) at the excitation wavelength of 485 nm and the emission wavelength of 535 nm.

### Gut microbiota (GM) study

#### Feces collection and fecal DNA extraction

The feces were collected aseptically from RD-fed 8-week-old mice raised under normal conditions (*n* = 8/genotype) and 16-week-old mice (8-week-old mice + 8 weeks of RD or WD feeding; *n* = 8/genotype/diet). Overall, 72 fecal samples were collected for sequencing. The mice were placed individually in clean cages with no food, water, and bedding for feces collection, and fecal pellets contaminated with urine or trampled by mice were not collected. The fecal samples were immediately frozen at −80 °C until DNA extraction. The fecal DNA was extracted using QIAamp DNA Stool Mini Kit (Qiagen, Cat. No. 51504, Germany) following the protocol specified by the manufacturer. The frozen fecal material was not allowed to thaw, and the lysis buffer was directly added to the frozen feces to avoid “freeze-thawing” before DNA extraction, which can potentially lead to sequencing bias [34].

#### 16S rRNA gene sequencing

The highly conserved regions of the 1500-bp length 16S rRNA gene are interspersed with 9 hypervariable regions (V1–V9) that contain information about higher or lower Linnaean taxonomic levels based on the degree of their variability [35]. Hypervariable regions, V3–V4, were amplified and sequenced using fecal DNA (a total of 72 DNA samples). The fecal DNA concentration was checked using a NanoDrop (Thermo Fisher Scientific, USA). The DNA concentration was normalized to 50 ng/μl in all the samples using 1X TE buffer (10 mM TRIS, pH 8 adjusted with HCl, 1 mM EDTA). A universal primer set, 341F (5′-CCTAYGGGRBGCASCAG-3′) and 802R (5′-TACNVGGGTATCTAATCC-3′), was used to amplify the hypervariable V3 and V4 regions of the microbial 16S rRNA gene. Each sample was amplified in three separate reactions, and the PCR products were pooled for further processing to minimize potential PCR bias. PCR product was purified and prepared for sequencing on an Illumina MiSeq sequencer as described previously [36]. The samples were sent to Novogene (Beijing, China) for sequencing on an Illumina MiSeq sequencer with a paired-end (PE) mode and a read length of 300 bp. The 16S rRNA gene PCR, data trimming, and processing protocols are described previously [36]. The quantitative insights into microbial ecology (QIIME) platform were used to process the raw data. The QIIME pipeline was used to annotate the operational taxonomic units (OTUs) and generate the relative abundance at different levels of Linnaean bacterial classification.

#### Metagenome sequencing and functional gene analysis

Metagenome analysis was carried out to study the functional gene changes in the GM due to Epac1 or Epac2 deletion with RD or WD. The fecal DNA samples from 16-week-old Epac1^–/–^, Epac2^–/–^ and WT mice in the WD study (8-week-old mice + 8 weeks of RD or WD feeding) from two separate batches were used for metagenome analysis. A composite DNA sample was prepared by pooling the fecal DNA from 6 mice in each group to achieve a DNA concentration of 100ng/ μl. Six composite samples (3 genotypes, 2 diets) were used for the metagenomics analyses. The DNA samples were sent to Beijing Genomics Institute (BGI) for shotgun metagenome sequencing. The raw metagenome datasets were cleaned, normalized, and processed as described previously [37]. Kyoto Encyclopedia of Genes and Genome (KEGG) database was used to annotate functional microbial proteins. The metagenome sequence coverage values were used to construct heatmaps using R software to visualize the changes in the functional gene coverage in specific pathways among different genotypes under normal or WD conditions.

#### Metagenome species profiling

Raw sequencing reads were assembled with MEGAHIT [38] using default parameters. Assembled contigs were annotated using Kraken2 [39] with the standard NCBI bacterial database. Raw reads were realigned to contigs using Bowtie2 [40]. Species abundance was calculated as the total read count aligned to each annotated species contig. Species with a relative abundance of more than 1% in at least 5% of the samples were kept for analysis and visualization.

### Western blot analysis

Radioimmunoprecipitation assay (RIPA) buffer was used to extract proteins from EWAT and jejunum. Protein concentration in the lysate was measured using a protein dye reagent (Bio-Rad, Cat. No. 500-0006, USA) as per the manufacturer’s instructions. The proteins were separated by 10% of sodium dodecyl sulfate-polyacrylamide gel electrophoresis (SDS-PAGE). Protein samples (25μg) and the protein marker were loaded in the wells and separated by SDS-PAGE at 70V for 3–4 h. The transfer was allowed to occur overnight in a cold room maintained at 4 °C, under 30V. The membranes were blocked with 5% of nonfat milk (Bio-Rad, Cat. #170-6404) in TBS-T (Tris-HCl, 50mM; NaCl, 150mM; Tween20, 0.1%), and immunoblotting was carried out using Epac1 (Cell Signaling #4155) and GAPDH (Abcam #9484) antibodies.

### Real-time (RT) quantitative polymerase chain reaction (qPCR)

Jejunum, EWAT and liver tissues were homogenized in liquid nitrogen, and RNA was extracted using TRI reagent (Molecular Research Center, Inc., USA) following the manufacturer’s protocol. The cDNA was prepared using PrimeScript™ RT Master Mix (Takara, Cat. #RR036A, Japan), and RT-qPCR was carried out using SYBR® Premix Ex Taq ™ II (Tli RNaseH Plus) (Takara Cat. #RR820, Japan) as per the manufacturer’s instructions. Primer sequences for the targeted mouse sequences are listed in Additional file 1: Table S1. The RT-qPCR was performed using Bio-Rad iQ5 real-time thermal cycler (Bio-Rad, USA) linked to iQ5 Optical System Software. The PCR was conducted using the following program: a 3-min initial denaturation at 95 °C followed by 40 cycles of denaturation at 95 °C for 15 s and amplification at 60 °C for 30 s. The cycle threshold (Ct) values of the experimental genes in the samples were normalized against the respective Ct values of the internal controls/ housekeeping genes, RPL19 in the WAT [5], and GAPDH in the jejunum and liver.

### Biochemical analysis

Serum lipocalin-2 (LCN2) concentrations were measured using an in-house ELISA kit. The details of the preparation of the kit contents and LCN2 ELISA are previously described [41]. Briefly, serum stored at −80 °C was thawed on ice and diluted (1:50) with 1× PBS. One hundred microliters of the diluted serum and the standards were applied to the wells of a 96-well microtiter plate coated with murine anti-LCN2 antibodies and incubated at 37 °C for 1 h. The plates were washed with PBS thrice before and after incubation with 100 μl of the detection antibody for 2 h and incubated with streptavidin-conjugated horseradish peroxide for 1 h, followed by incubation with tetramethyl-benzidine reagent for 15 min. Finally, the reaction was stopped with 100 μl of 2mol/l H_2_SO_4_, and the absorbance at 450nm was measured.

### Bioinformatic and statistical analysis

Graphpad Prism 9.2.0 software (GraphPad Software Inc., CA, USA) was used to perform the statistical analyses and visualize the data. The statistical significance was determined by two-way ANOVA, one-way ANOVA, or Student’s *t* test unless otherwise specified. Shannon’s diversity indices were calculated using the R package Vegan [42]. Partial least squares discriminatory analysis (PLS-DA) was performed as a supervised multivariate dimensionality reduction tool to visualize microbial features that best explain changes in the GM across groups. PLS-DA was implemented through the “plsda” function from the R package mixOmics [43]. Heatmaps were constructed with R software using the packages ggplot2 [44] and pheatmap [45] to visualize 16S rRNA gene sequencing and metagenomics data. The R scripts used for the bioinformatics analyses are detailed in Additional file 2.

## Results

### The Epac2^–/–^ mice have gut dysbiosis under physiological conditions

**Fig. 1:**
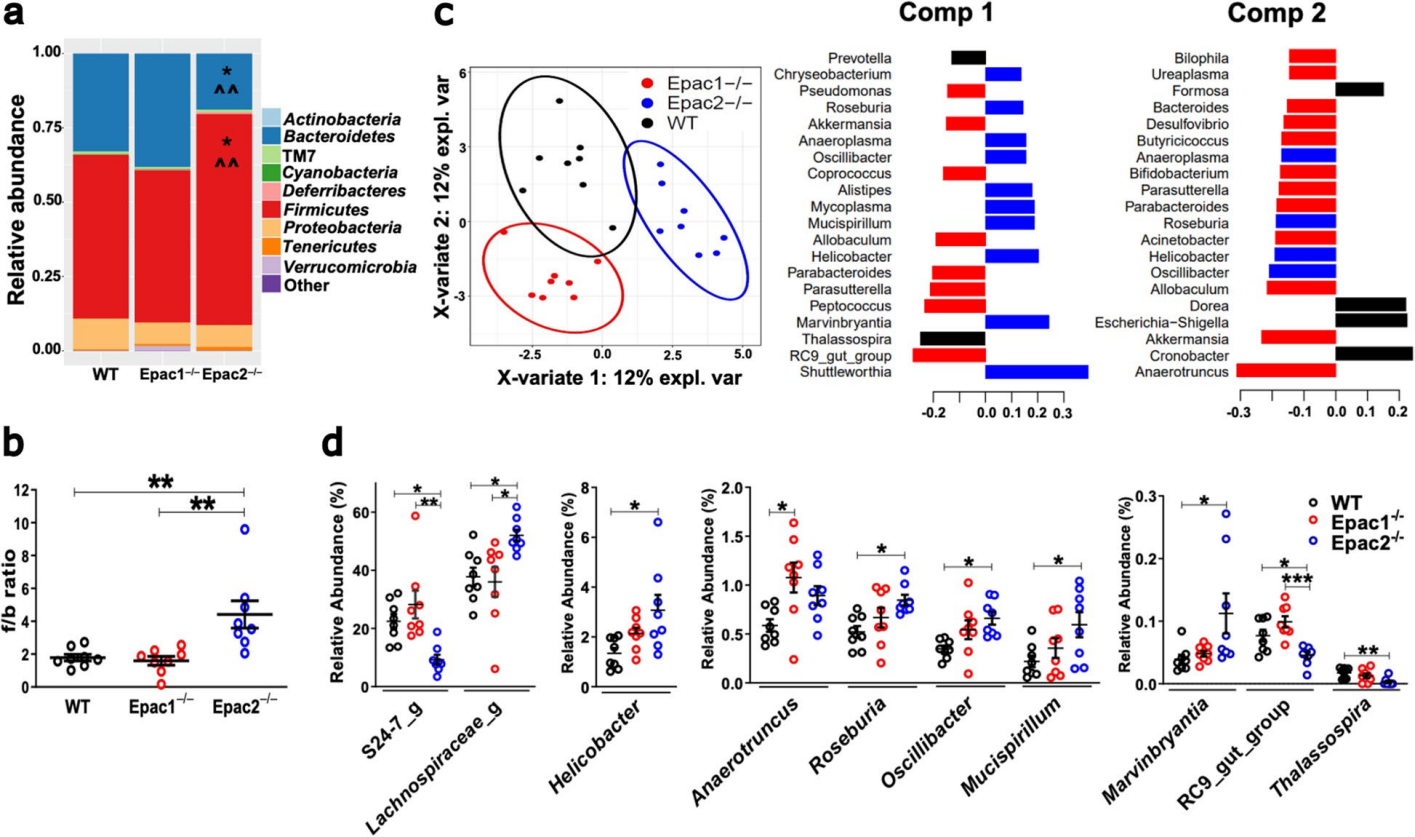
Characterization of baseline GM of WT, Epac1^–/–^, and Epac2^–/–^ mice under physiological conditions. All data are from 8-week-old mice fed the RD**.** 16S rRNA gene sequencing data were used to determine the abundance of bacterial taxa (*n* = 8/genotype). **a** Stacked barplots showing the mean relative abundance of the top 10 bacterial phyla detected in each genotype. **b** Scatterplots showing that Epac2^–/–^ mice had a significantly higher f/b ratio compared to WT and Epac1^–/–^ mice. **c** PLS-DA scatterplot showing distinct baseline GM in different genotypes. Axis title details the percentage of variation explained by each dimension. **d** Scatterplots showing the relative abundance of bacterial genera with significant differences between the genotypes; for the sake of clarity, the genera were plotted in separate graphs. The black, red, and blue colors represent WT, Epac1^–/–^, and Epac2^–/–^ mice, respectively (**b–d**). Data are presented as mean (**a**) and mean ± SEM (**b, d**). Statistical analyses were performed by one-way ANOVA with Tukey’s post hoc test, *P<0.05, **P<0.01, ***P<0.001; (**a**) *P<0.05 vs. WT and ^^*P*<0.01 vs. Epac1^−/−^ mice

To investigate if Epac1 or Epac2 deficiency in mice led to GM imbalance under physiological conditions, we characterized the GM of 8-week-old RD-fed WT, Epac1^–/–^, and Epac2^–/–^ mice raised under normal conditions. The 16S rRNA gene sequencing results showed a total of 41 bacterial phyla, 89 classes, 183 orders, 339 families, and 637 genera (Additional file 3: Table S2, Additional file 4: Table S3, Additional file 5: Table S4, Additional file 6: Table S5, Additional file 7: Table S6). Consistent with previously reported mouse gut microbiota characterization [7], our sequencing results indicated that *Firmicutes* and *Bacteroidetes* were the most predominant phyla detected, followed by *Proteobacteria* (Fig. 1a). The Epac2^–/–^ mice had significantly lower *Bacteroidetes* and higher relative abundance of *Firmicutes* (Fig. 1a), and their representatives at different Linnaean hierarchal levels (Additional file 8: Fig. S1a-b, S2a-b) under normal conditions. Consistently, the f/b ratio was significantly higher in Epac2^–/–^ mice, suggesting gross GM imbalance in these mice under physiological conditions (Fig. 1b). In addition, compared to the WT mice, the GM of Epac2^–/–^ mice was significantly enriched in classes, *Epsilonproteobacteria* and *Deferribacteres*, and their representative orders and families, *Campylobacteriales*, *Deferribacterales*, *Helicobacteraceae*, and *Deferribacteraceae* (Additional file 8: Fig. S1a-b, S2c-d). The Shannon’s diversity index showed that the GM diversity was similar in the three genotypes (Additional file 8: Fig. S2e), but PLS-DA demonstrated that Epac1^–/–^ and Epac2^–/–^ mice populations could be clearly distinguished from WT mice (Fig. 1c). However, most significant differences in the abundance of various genera, including enrichment of *Mucispirillum*, *Roseburia*, *Oscillibacter*, *Marvinbryantia*, and *Helicobacter*, and reduced RC9 gut group and *Thalassospira* were detected in Epac2^–/–^ mice (Fig. 1d) corroborating gross imbalance in baseline GM in Epac2^–/–^ mice seen at higher taxonomic levels. Unlike Epac2^–/–^ mice, a few significant differences were detected in the GM of Epac1^–/–^ versus WT mice, such as an increased abundance of *Anaerotruncus* (Fig. 1d), *Erysipelotrichi* and its representative order and class (Additional file 8: Fig. S1a-b, S2b). Collectively, these data show that both Epac1^–/–^ and Epac2^–/–^ mice fed the RD harbored distinct GM compared to WT mice, although more significant gut dysbiosis was detected in Epac2^–/–^ mice under physiological conditions.

### An 8-week WD caused a significant shift in the GM composition regardless of genotype and Epac1 deficiency modestly exacerbated the GM dysbiosis induced by WD

**Fig. 2:**
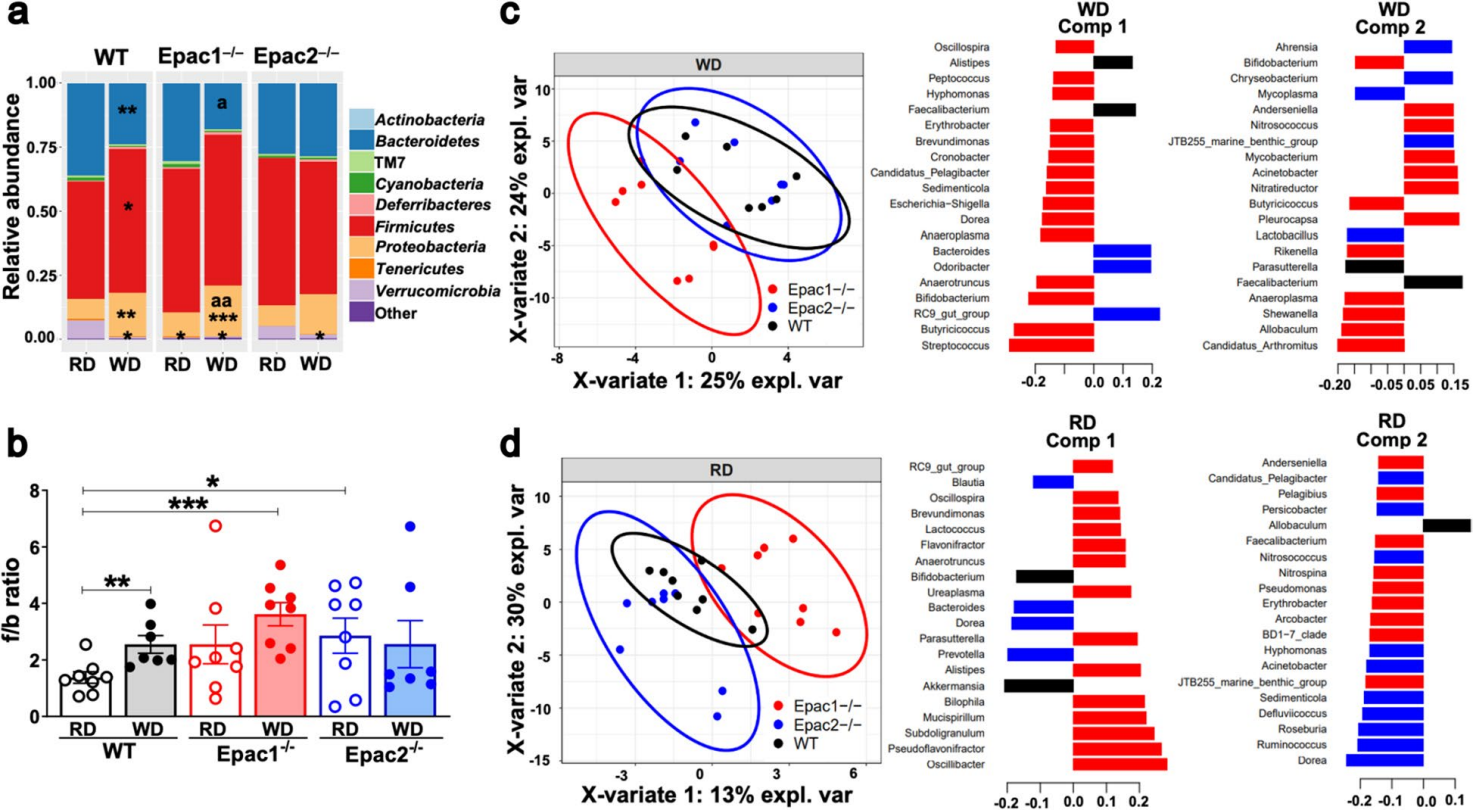
Alterations in GM composition induced by 8-week WD in WT, Epac1^–/–^, and Epac2^–/–^ mice. All data are from 16-week-old mice (8-week-old + RD or WD for 8 weeks)**.** 16S rRNA gene sequencing data were used to determine the abundance of bacterial taxa (*n* = 7–8/genotype/diet). **a** Stacked barplots showing the alterations in the relative abundance of the top 10 bacterial phyla in the RD- and WD-fed groups of each genotype. **b** f/b ratio in the 6 groups (3 genotypes × 2 diets). **c,d** PLS-DA scatterplot of genotypes fed WD (**c**) or RD (**d**) at 16 weeks old and respective loading vector bar plots for the two components. The top twenty variables with the highest mean contribution to each component are plotted. The color corresponds to the genotype the genera is found most abundant in. Data are presented as mean (**a**) and mean ± SEM (**b**). Statistical significance assessed by unpaired Student’s *t* test, *P<0.05, **P<0.01, ***P<0.001 vs. RD-fed WT control mice, and ^a^*P*<0.05, ^aa^*P*<0.01 vs. RD-fed Epac1^**–/–**^ mice

The 16S rRNA gene sequencing data showed that WD feeding caused an increase in *Firmicutes* and *Proteobacteria*, and decrease in *Bacteroidetes* and *Verrucomicrobia* in the WT mice compared to their RD-fed counterparts (Fig. 2a). Numerous studies have reported that HFD and obesity cause a distinct shift in the bacterial composition characterized by increased *Firmicutes* and decreased abundance of *Bacteroidetes* [5, 15, 16]. Consistently, our sequencing results showed that WD caused a significant increase in the f/b ratio in WD-fed WT mice compared to their RD-fed WT control mice (Fig. 2b). However, WD feeding did not result in a significantly higher f/b ratio in Epac1^–/–^ and Epac2^–/–^ mice than their respective RD-fed groups (Fig. 2b). This may be owning to a trend of higher f/b ratio or significantly higher f/b ratio detected in RD-fed Epac1^–/–^ and Epac2^–/–^ mice, respectively (Fig. 2b). An 8-week WD significantly enriched the representatives of phylum *Proteobacteria, Deltaproteobacteria*, *Desulfovibrionales*, and *Desulfovibrionaceae* (Additional file 8: Fig. S3a-b, S4c), and reduced S24-7 and *Verrucomicrobiaceae* (Additional file 8: Fig. S4b,d) in mice regardless of genotypes. These data corroborate previous findings that the gut dysbiosis is characterized by significant alterations in *Proteobacteria*, which have been associated with various metabolic and intestinal diseases [46]. There were no significant differences in the GM composition among WD-fed WT, Epac1^–/–^, and Epac2^–/–^ mice (Fig. 2a,b, S3a-b, S4a-d). However, when compared with RD-fed WT control, WD-fed Epac1^–/–^ showed a more significant increase in f/b ratio and Proteobacteria abundance than WD-fed WT mice (Fig. 2a,b). Furthermore, PLS-DA showed that WD feeding caused a similar shift in the GM composition of WT and Epac2^–/–^ mice, but the GM composition of corresponding Epac1^–/–^ mice was distinct (Fig. 2c). Taken together, these data showed that Epac1 deficiency modestly augmented WD-associated gut dysbiosis in mice. Additionally, the GM communities of 16-week-old Epac1^–/–^ mice fed the RD could be clearly distinguished from corresponding WT and Epac2^–/–^ mice (Fig. 2d).

**Fig. 3:**
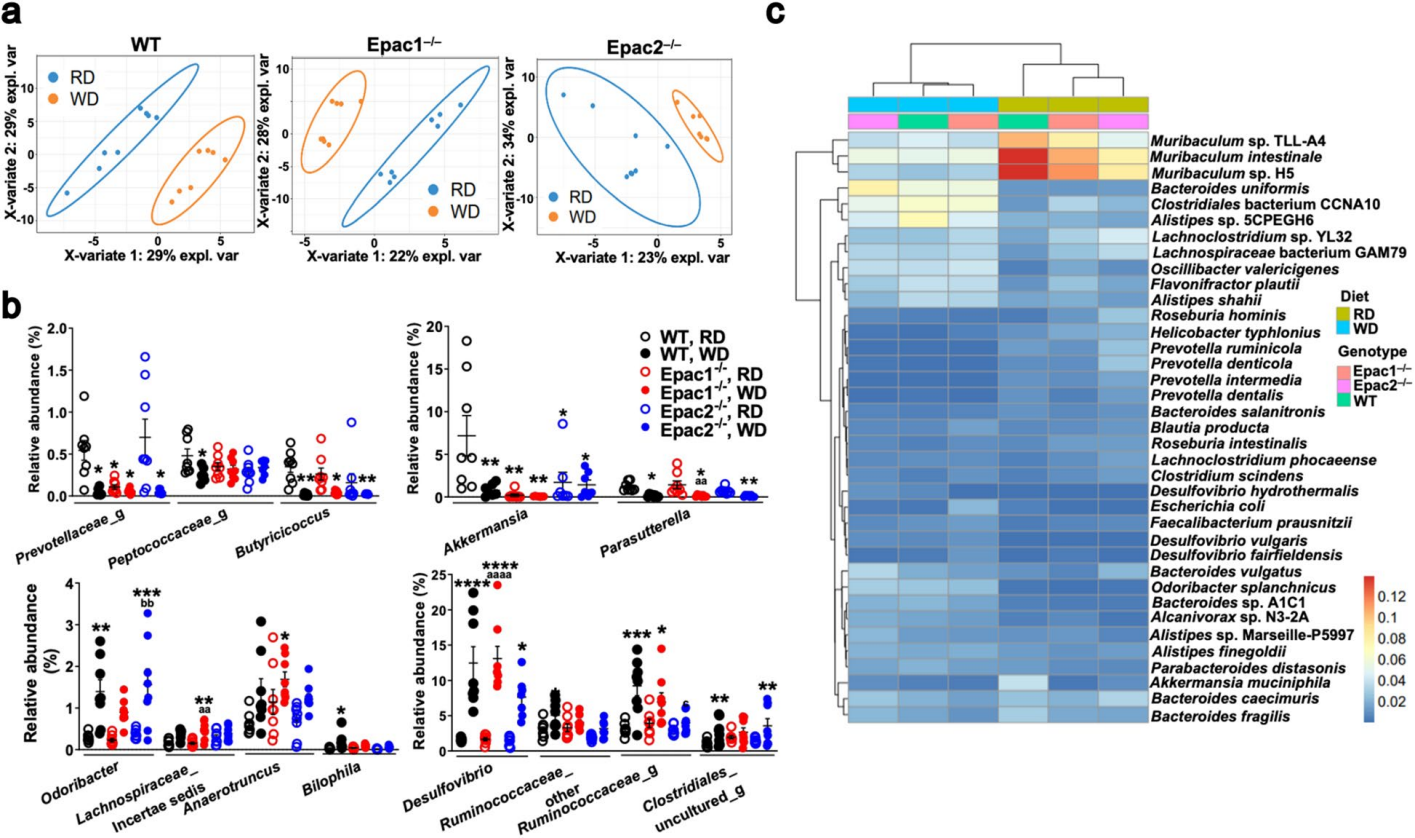
Significant alterations in GM composition at genus and species levels due to WD feeding or Epac1/ Epac2 deficiency. All data are from 16-week-old mice (8-week-old + RD or WD for 8 weeks) (*n* = 7–8/diet/genotype). **a** PLS-DA scatterplots showing that WD feeding caused a marked shift in GM communities in WT, Epac1^–/–^, and Epac2^–/–^ compared to their respective RD-fed groups. Axis title details the percentage of variation explained by each dimension. **b** Scatterplots showing the relative abundance of bacterial genera with significant differences between the 6 groups (3 genotypes × 2 diets); for the sake of clarity, the genera were plotted in separate graphs. Data are presented as mean ± SEM and statistical significance was analyzed by two-way ANOVA with Tukey’s post hoc test, *P<0.05, **P<0.01, ***P<0.001, ****P<0.0001 vs. RD-fed WT mice; ^aa^*P*<0.01, ^aaaa^*P*<0.0001 vs. RD-fed Epac1^–/–^ mice; ^bb^*P*<0.01 vs. RD-fed Epac2^–/–^ mice, ^c^*P*<0.05 vs. WD-fed WT mice. **c** Relative abundance heatmap of predominant bacterial species and changes in species abundance detected due to WD feeding or Epac1/ Epac2 deficiency in a shotgun analysis performed using a composite DNA sample of fecal DNA pooled from 6 mice in each of the six groups (3 genotypes × 2 diets). The colored tiles in the body of the heatmaps indicate the relative abundance of the bacterial species; the scalebar is alongside the heatmap

The WD caused a clear shift in the GM composition within the three genotypes when compared to their respective RD-fed counterparts (Fig. 3a). A significant enrichment of *Odoribacter*, *Desulfovibrio*, *Bilophila*, and two uncharacterized genera in *Ruminococcaceae*; reduction of *Akkermansia*, *Parasutterella*, *Butyricicoccus*, and an uncharacterized genus in *Prevotellaceae* and *Peptococcaceae* each was detected in WD-fed WT animals compared to their RD-fed controls (Fig. 3b). Significant positive correlation of *Desulfovibrio*, and negative correlation of *Akkermansia*, *Parasutterella*, *Butyricicoccus*, and an uncharacterized genus in *Prevotellaceae* with WD feeding was consistently detected across genotypes (Fig. 3b). Moreover, RD-fed Epac1^–/–^ and Epac2^–/–^ mice were detected with significant negative correlation with *Akkermansia*, and an uncharacterized genus in *Prevotellaceae* was significantly reduced in RD-fed Epac1^–/–^ mice compared to RD-fed WT mice (Fig. 3b).

Shotgun sequencing using a composite DNA sample of the 6 different groups (3 genotypes × 2 diets; Additional file 9: Table S7) showed that four *Prevotella* species, *P. ruminicola*, *P. denticola*, *P. intermedia*, and *P. dentalis*, and *Helicobacter typhionius* were consistently reduced in the WD-fed mice of the three genotypes compared to the RD-fed mice (Fig. 3c). Conversely, *Odoribacter splanchnicus*, *Alistipes shahii*, *Bacteroide*s sp. AIC1, and *Desulfovibrio vulgaris* were enriched in the WD-fed groups compared to the RD-fed mice of all the genotypes (Fig. 3c). The most noteworthy was conspicuously reduced *Muribaculum* sp. TLLA4, *Muribaculum intestinale, Muribaculum* sp. H5, *Akkermansia muciniphila*, and *Bacteroides fragilis* in WD-fed mice of all genotypes, as well as a reduced abundance of these species in RD-fed Epac1^–/–^ and Epac2^–/–^ mice compared to the RD-fed WT mice (Fig. 3c). Collectively, these data suggested that WD feeding or Epac1/ Epac2 deficiency in RD-fed mice led to GM imbalances in mice.

### WD feeding in WT mice or Epac1/ Epac2 deficiency caused alterations in the functional metagenome in mice

The analysis of the metagenome has been instrumental in understanding the microbial ecosystems in various habitats and their potential function [47]. Shotgun metagenomic sequencing revealed 7903 genes, which were annotated with KEGG numbers (Additional file 10: Table S8, Additional file 11: Table S9). The WD-fed groups of WT, Epac1^–/–^, and Epac2^–/–^ mice clustered together, suggesting that WD drove the shift in the functional metagenome regardless of genotype (Additional file 8: Fig. S5). An 8-week WD caused an upregulation of genes belonging to KEGG pathways, “two-component system,” “cell growth,” “bacterial motility proteins,” “bacterial secretion system,” “secretion system,” “membrane transport,” “nucleotide metabolism,” and “amino acid metabolism” in mice regardless of genotype compared to the RD-fed WT mice (Additional file 8: Fig. S6). The downregulated pathways included “pentose and glucuronate interconversions”, “ubiquinone and other terpenoid-quinone biosynthesis,” and “lipopolysaccharide biosynthesis” (Additional file 8: Fig. S6). In particular, RD-fed Epac1^–/–^ and Epac2^–/–^ mice had upregulation of several pathways, including “two-component system,” “bacterial motility proteins,” “secretion system,” and “cell growth” (Additional file 8: Fig. S6), which had been altered due to WD feeding either in our study or previously reported studies [48, 49], suggesting that deficiency of Epac1 or Epac2 in RD-fed mice was associated with similar changes in the gut microbiome as induced by WD feeding.

### WD-fed Epac1^–/–^ mice develop more severe obesity and impaired oral glucose tolerance

**Fig. 4:**
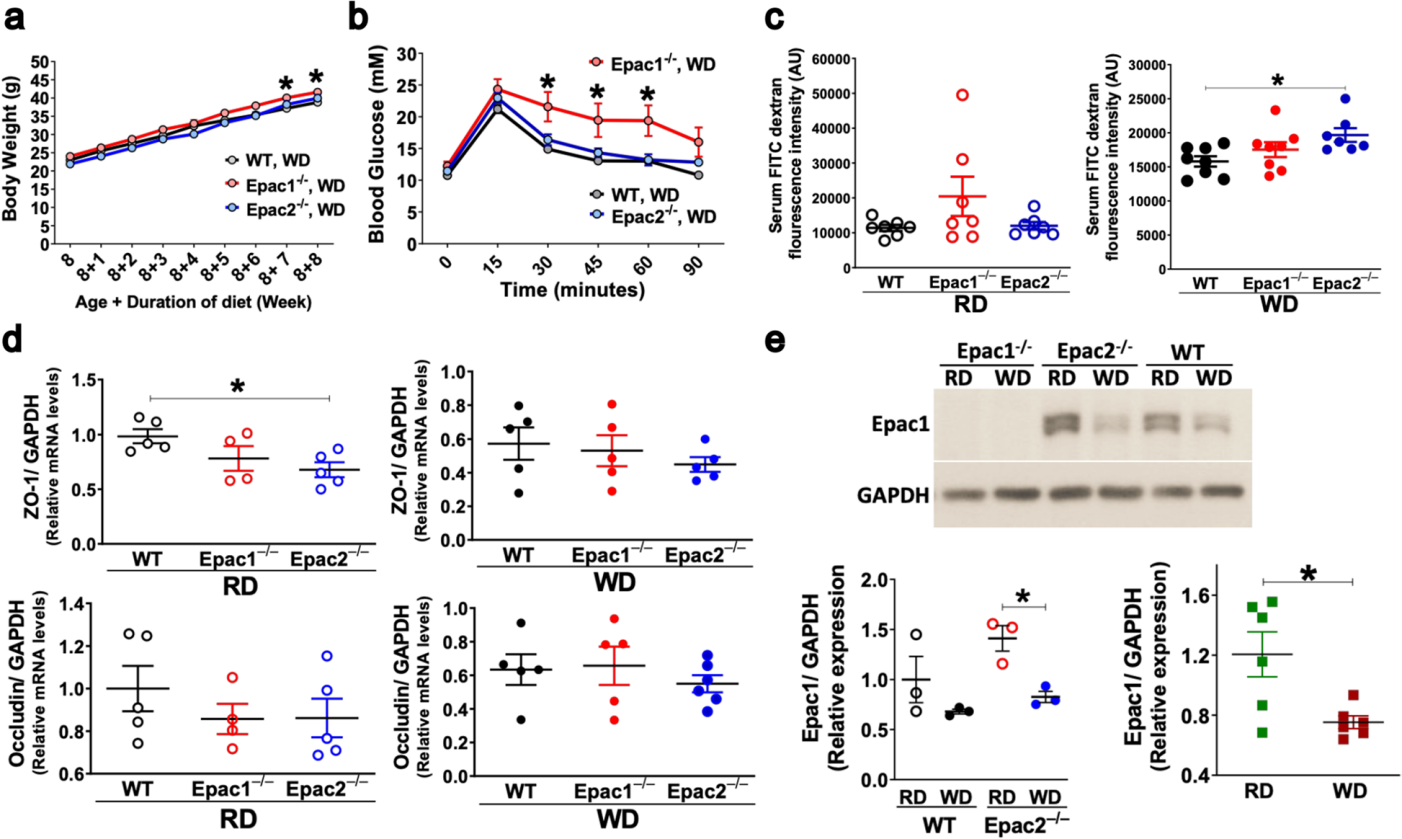
Obesity-related phenotypes and gut permeability changes following 8-week WD. All mice were 16-week-old (8-week-old + RD or WD for 8 weeks). **a** Body weight of WT, Epac1^–/–^, and Epac2^–/–^ mice while feeding WD over 8 weeks (*n* = 9–11/genotype/diet). **b** Blood sugar concentrations in WT, Epac1^–/–^, and Epac2^–/–^ mice during an oral glucose tolerance test (OGTT) (*n* = 8–9/genotype/diet). **c** In vivo gut permeability test (*n* = 7–8/genotype/diet); RD-fed mice (left panel) and WD-fed mice (right panel); AU, arbitrary unit. **d** Scatterplots showing relative mRNA levels of jejunal cell-cell TJ markers, ZO-1 (top panels), and occludin (bottom panels) (*n* = 4–6/genotype/diet). **e** Representative Western blot image showing Epac1 protein expression was detected in the jejunum of RD and WD-fed WT and Epac2^–/–^ mice, but not Epac1^–/–^ mice (top panel). Scatterplots showing quantification of Epac1 protein expression in the jejunum of WT and Epac2^–/–^ mice (*n* = 3/genotype/diet) (bottom left panel), and all mice regardless of genotype fed RD or WD (*n* = 6/diet) (bottom right panel). All data are presented as mean ± SEM. Statistical significance was assessed by two-way ANOVA with Tukey’s post hoc test (**a**, **b**); one-way ANOVA with Tukey’s post hoc test and unpaired Student’s *t* test for comparison between 3 and 2 groups, respectively, *p<0.05 (**c–e**)

Previously, we have reported that Epac1^–/–^ mice developed more severe obesity, impaired oral glucose tolerance, and insulin resistance when fed the HFD for 8 weeks [26]. In the present study, 8-week-old WT, Epac1^–/–^ and Epac2^–/–^ mice were randomized to either RD or WD for 8 weeks. The WD-fed Epac1^–/–^ mice weighed heavier compared to their counterpart WT mice with significantly greater body weight after feeding WD for 7 and 8 weeks (Fig. 4a). The WD-fed mice of the three genotypes had similar blood glucose, which was significantly higher than their respective RD-fed controls (Additional file 8: Fig. S7). The WD-fed Epac1^–/–^ mice had significantly lower oral glucose tolerance than the corresponding WT and Epac2^–/–^ mice (Fig. 4b). However, the Epac2^–/–^ mice fed the WD displayed similar OGTT to WD-fed WT mice (Fig. 4b).

### The WD feeding led to a greater increase in the gut permeability in Epac2^–/–^ mice

Apical tight junction (TJ) proteins are critical in maintaining intestinal epithelial barrier function and paracellular permeability. Alternation of tight junction proteins leads to epithelial barrier dysfunction and causes inflammation associated with the pathogenesis of diet and GM-induced metabolic complications [5, 6, 50, 51]. In agreement with previous findings [5], WD feeding led to a significantly greater increase in the gut permeability in the WT and Epac2^–/–^ mice than the RD-fed counterparts (Additional file 8: Fig. S8a). In particular, a significant increase in gut permeability was detected in the WD-fed Epac2^–/–^ mice than the WD-fed WT mice (Fig 4c). Reduced expression of tight junction proteins, ZO-1 and occludin, have been reported to underlie the gut permeability alterations [5]. Therefore, we determined ZO-1 and occludin mRNA expression in the middle segment (jejunum) of the small intestine. Consistent with the in vivo gut permeability test results, the ZO-1 and occludin mRNA expression was significantly lower in the jejunum of WD-fed WT and Epac2^–/–^ mice than their respective RD-fed counterparts (Additional file 8: Fig. S8b). RD-fed Epac2^–/–^ mice had a significantly lower expression of ZO-1 mRNA than corresponding WT mice in the jejunum (Fig. 4d), whereas a trend of reduced ZO-1 and occludin mRNA was observed in WD-fed Epac2^–/–^ mice than the corresponding WD-fed WT mice (Fig. 4d). On the other hand, no significant differences in the gut permeability, and ZO-1 and occludin mRNA levels were detected in the jejunum of WD-fed Epac1^–/–^ mice compared to their RD-fed counterparts (Additional file 8: Fig. S8a-b). Since Epac1 is the main Epac isoform expressed in the gut [52], we investigated if WD feeding altered Epac1 protein expression in the jejunum. The Western blot results showed that Epac1 protein expression was significantly lower in the jejunum of WD-fed mice than the RD-fed mice (Fig. 4e). Taken together, these data suggest that both Epac1 and Epac2 may have a role to play in regulating intestinal gut permeability.

### Epac1 deficiency led to more severe inflammation and macrophage infiltration in the white adipose tissue

**Fig. 5:**
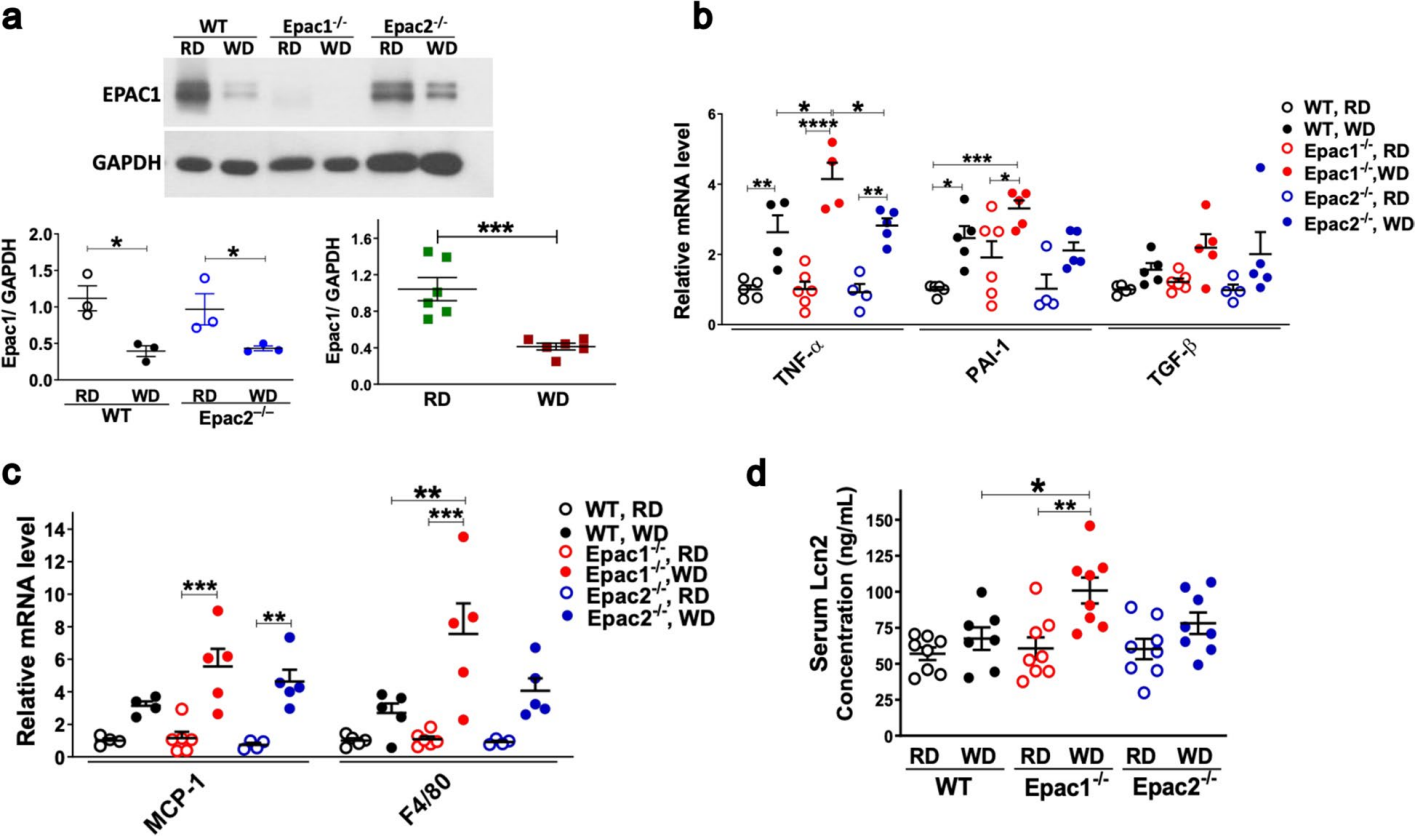
8-week WD reduces Epac1 protein expression in the EWAT, and Epac1 deficiency in mice exacerbates inflammation. All mice were 16-week-old (8-week-old + RD or WD for 8 weeks). **a** Representative Western blot image showing Epac1 protein expression was detected in the EWAT of RD and WD-fed WT and Epac2^–/–^ mice, but not Epac1^–/–^ mice (top panel). Scatterplots showing quantification of Epac1 protein expression in the EWAT of WT and Epac2^–/–^ mice (*n* = 3/genotype/diet) (bottom left panel), and all mice regardless of genotype fed RD or WD (*n* = 6/diet) (bottom right panel). Data are presented as mean ± SEM. Statistical significance was assessed by unpaired Student’s *t* test. **b,c** RT-qPCR analysis of mRNA concentrations of **b** pro-inflammatory markers, TNF-α, PAI-1, and TGF-β. **c** Macrophage infiltration markers, MCP-1, F4/80 (*n* = 4–6/genotype/diet). **d** Serum LCN2 protein concentration (*n* = 7–8/genotype/diet). All data are presented as mean ± SEM. Statistical significance was assessed by two-way ANOVA with Tukey’s post hoc test, *P<0.05, **P<0.01, ***P<0.001, ****P<0.0001

White adipose tissue (WAT) is a hub of inflammatory events in the diet-induced metabolic syndrome [53]. Under HFD conditions, WAT secrete various cytokines that trigger increased recruitment of macrophages, which enhances the production and dissemination of pro-inflammatory markers leading to insulin resistance [54]. Since Epac1 but not Epac2 is reported to be expressed in the WAT [55], we investigated if Epac1 protein expression was altered in the epididymal white adipose tissue (EWAT) under the influence of WD. The results showed that Epac1 protein expression was significantly lower in the EWAT of WT and Epac2^–/–^ mice after feeding WD for 8 weeks than the RD-fed mice (Fig. 5a). To investigate if Epac1 and/or Epac2 mediate the cascade of inflammatory events in the WAT triggered by WD, we conducted RT-PCR to analyze a range of pro-inflammatory (TNF-α, PAI-1, TGF-β), macrophage infiltration (MCP-1, F4/80), and oxidative stress (STAMP2, NAPDHox) markers in RD and WD-fed mice. The relative mRNA levels of TNF-α, PAI-1, MCP-1, and F4/80 were significantly increased in the EWAT after 8 weeks of WD feeding in the WT, Epac1^–/–^, and Epac2^–/–^ mice (Fig. 5b,c). The WD-fed Epac1^–/–^ had a significantly greater increase in TNF-α and F4/80 mRNA expression in the EWAT than the corresponding WD-fed WT mice (Fig. 5b,c), suggesting that deficiency of Epac1 led to more severe inflammation through increased macrophage infiltration and pro-inflammatory TNF-α in the WAT upon WD feeding. Additionally, there was an insignificant trend of increased STAMP-2 and NADPH-ox, in the EWAT of WD-fed WT, Epac1^–/–^, and Epac2^–/–^ mice (Additional file 8: Fig. S9a).

### Epac1 deficiency in mice led to significantly increased circulating lipocalin-2 (LCN2) concentration after WD feeding

The pro-inflammatory cytokine, TNF-α, is reported to mediate the signaling of two key adipokines, lipocalin2 (LCN2) and adiponectin [56, 57]. A significantly lower adiponectin protein expression in the plasma and lower adiponectin mRNA levels in the WAT were detected in obese humans, which were inversely correlated with TNF-α [57]. First, we performed RT-qPCR to determine if Epac1 or Epac2 deficiency led to altered adiponectin and LCN2 expression in the liver and the WAT. The adiponectin mRNA expression was decreased in the liver and the EWAT of the WD-fed WT, Epac1^–/–^, and Epac2^–/–^ mice compared to their RD-fed counterparts, with no significant differences between the genotypes in the RD- or WD-fed groups (Additional file 8: Fig. S9b). Conversely, the LCN2 mRNA expression was significantly higher in both liver and EWAT of the WD-fed WT and Epac2^–/–^, but not Epac1^–/–^, mice than that in their respective RD-fed counterparts (Additional file 8: Fig. S9c). However, a trend of increased LCN2 mRNA was noted in RD-fed Epac1^–/–^ mice in both liver and EWAT (Additional file 8: Fig. S9c). Recently, it was shown that LCN2 is positively correlated with insulin resistance and inflammation in humans [58]. We found that circulating serum LCN2 protein was significantly higher in the serum of WD-fed Epac1^–/–^ mice than that of the corresponding WD-fed WT mice (Fig. 5d), suggesting greater systemic inflammation in Epac1^–/–^ mice. However, no significant differences were noted in LCN2 levels among the WD-fed WT and Epac2^–/–^ mice and their corresponding RD-fed mice. These results suggest that Epac1 deficiency may have expedited the induction of serum LCN2 compared to the other two genotypes.

## Discussion

The GM dysbiosis is a crucial driver of obesity [4–8]. An increase in f/b ratio, by and large, is associated with several metabolic diseases in humans and animal models, such as obesity [5, 7, 8], diabetes [59], and nonalcoholic steatohepatitis [60], barring some conflicting reports of GM enriched in Bacteroidetes and reduced Firmicutes in metabolic diseases [12, 61]. As shown in Figs. 1b and 2b, RD-fed Epac2^–/–^ mice had significantly higher f/b ratio at ages 8 and 16 weeks old, suggesting GM dysregulation in these mice under normal conditions. Despite PLS-DA identifying multivariate genera signatures that can clearly distinguish populations of Epac1- and Epac2-deficient mice from WT mice, significant differences in individual genus abundance were noted mainly in Epac2-deficient mice. The Epac2^–/–^ mice GM was characterized by significant alterations in several taxa which have been previously associated with metabolic diseases, including *Mucispirillum* [62], *Oscillibacter* [63], *Marvinbryanti*a [64], *Helicobacter* [65], RC9 gut group [66], *Thalassospira* [67] and *Epsilonbacteria* [68]. Collectively, 16S rRNA gene sequencing data demonstrated that Epac2^–/–^ mice were associated with gut dysbiosis under physiological conditions. These results were surprising since no obvious metabolic defects have been noted in Epac2^–/–^ mice under physiological conditions, although we have reported that Epac2^–/–^ mice suffer from anxiety, depression, and defects in neurogenesis under physiological conditions [69]. It remains unclear if the dysregulation of GM detected in these mice under normal conditions is linked to their depressive-like phenotype.

To investigate if WD diet feeding exacerbated GM dysbiosis in Epac1^–/–^ or Epac2^–/–^ mice compared to the WT mice, we characterized the GM of these mice again after feeding WD or RD for 8 weeks. The 16S rRNA gene sequencing data showed that WD caused a significant dysbiosis evident at various Linnaean classification levels in WT, Epac1^–/–^, and Epac2^–/–^ mice. However, no significant differences were detected in the abundance of various taxa between WD-fed WT, Epac1^–/–^, and Epac2^–/–^ mice. Despite substantially dysregulated baseline GM, WD-fed Epac2^–/–^ mice did not exhibit more severe gut dysbiosis compared to WD-fed WT mice. In particular, WD feeding did not lead to a more significant increase in f/b ratio in Epac2^–/–^ mice. However, the 16-week-old Epac2^–/–^ mice fed the RD did maintain a significantly higher f/b ratio as seen at 8 weeks. On the other hand, deficiency of Epac1 modestly augmented an increase in f/b ratio and *Proteobacteria* abundance in mice despite no significant differences in the majority of taxa comprising the baseline GM of 8-week-old Epac1-deficient mice compared to corresponding WT mice. Altogether, these data suggest that baseline GM composition did not predict the severity of gut dysbiosis after WD feeding in Epac-mutated mice.

The class *Deltaproteobacteria* and its representatives [49, 70] at different taxonomic ranks were significantly enriched in the WD-fed mice across genotypes in our study. Members of *Deltaproteobacteria* are known to reduce sulfate and produce LPS, which underlies the pathogenicity of these bacteria in animal models of diet-induced metabolic complications [70]. Indeed, GM enriched in *Proteobacteria* has been associated with several metabolic diseases, such as HFD-induced obesity [48, 49, 70], diabetes [12], and colitis [71]. The shotgun sequencing showed that *Desulfovibrio* species, *D. vulgaris*, was enhanced in WD-fed mice of all the genotypes, whereas *D. fairfieldensis* and *D. hydrothermalis* were differentially enhanced in WD-fed Epac1^–/–^, and WT mice and Epac2^–/–^ mice, respectively. On the other hand, taxa with significant negative correlations with WD across genotypes included *Verrucomicrobia* and its representatives at various taxonomic levels, S24-7, *Butyricicoccus*, *Parasutterella*, and an uncharacterised genus in *Prevotellaceae*. Our shotgun sequencing data showed that species belonging to the genera, *Akkermansia* (*A. muciniphila*), *Prevotella* (*P. ruminicola*, *P. denticola*, *P. intermedia*, and *P. dentalis*), and *Muribaculum* (*M. intestinale*, *M.* sp. H5, and *M.* TLL-A4) were consistently reduced in WD-fed WT, Epac1^–/–^, and Epac2^–/–^ mice compared to the RD-fed WT mice. The most notable species of *Verrucomicrobia*, *A. muciniphila*, has been reported to safeguard the gut epithelium barrier and reduce inflammation and glucose intolerance in mice [72, 73]. In addition, *Prevotella* has been associated with the fermentation of complex carbohydrates and the production of anti-inflammatory SCFAs, which play a crucial role in safeguarding the gut barrier [72–74]. The genus *Muribaculum* belongs to the family S24-7, which has been negatively correlated with HFD in previous studies [6].

As shown in Fig. 1d and Additional file 8: Fig. S2f, no significant differences were seen in the relative abundance of the majority of genera between 8-week-old Epac1^–/–^ and WT mice fed the RD, while corresponding Epac2^–/–^ mice were associated with significant alterations in several bacterial genera. However, at 16 weeks, Epac1^–/–^ and Epac2^–/–^ mice were detected with significant differences in the abundance of some noteworthy bacterial taxa compared to the corresponding WT mice. Most notably, 16S rRNA gene sequencing data showed that the genus, *Akkermansia*, was significantly lower in WD-fed mice of all the genotypes as well as RD-fed Epac1^–/–^ and Epac2^–/–^ mice than that in the RD-fed WT mice, as shown in Fig. 3b. Similarly, WD feeding was associated with significantly reduced abundance of the family S24-7 and a trend of reduced S24-7 was also noted in RD-fed Epac1^–/–^ and Epac2^–/–^ mice compared to that of the corresponding WT mice as shown in the Additional file 8: Fig. S4b. Consistent with the 16S rRNA gene sequencing results, species belonging to the genus *Akkermansia*, *A. Muciniphila*, and family S24-7, *M. intestinale*, *M.* sp. H5, were also reduced in RD-fed Epac1^–/–^, and Epac2^–/–^ mice. Furthermore, metagenome analysis of functional genes showed that several KEGG pathways which were upregulated in WD-fed mice in our study or in previous studies [48, 49], including “two-component system,” “secretion system,” “cell growth,” and “bacterial motility proteins” were upregulated in RD-fed Epac1^–/–^ and Epac2^–/–^ mice compared to those in the RD-fed WT mice. Taken together, WD was a crucial driver of gut microbiome dysregulation regardless of genotype, and deficiency of Epac1 or Epac2 also led to imbalances in the gut microbiome under RD-fed conditions.

We were the first to report that Epac1-deficient mice weigh significantly heavier on HFD with more severe insulin resistance and pancreatic islet hypertrophy [26]. Consistent with the HFD-fed Epac1^–/–^ mice [26], WD-fed Epac1^–/–^ mice weighed significantly heavier and developed a more severe glucose intolerance than the corresponding WT mice in the present study. Conversely, Yan et al. had reported that global Epac1 knockout mice were resistant to HFD-induced obesity and glucose intolerance and displayed enhanced leptin sensitivity [75]. Subsequently, Hu et al. reported that mice with Epac1 knocked out in the adipose tissue weighed significantly heavier and had a worse oral glucose intolerance due to impaired leptin signaling under HFD conditions [55]. Moreover, Epac2^–/–^ mice weighed significantly higher and developed more severe intraperitoneal glucose intolerance upon feeding a HFD due to enhanced leptin resistance [76]. Though we noted a significantly increased weight gain in WD-fed Epac2^–/–^ mice, the oral glucose intolerance in Epac2^–/–^ mice was similar to that in the corresponding WT mice after feeding WD for 8 weeks. The inconsistency in BW and glucose intolerance in various studies may be owning to differences in the fat content of the diets, the age of HFD initiation, duration of HFD feeding, glucose tolerance test techniques (OGTT versus IPGTT), and generation of KO mice with different approaches in deletion of Epac gene [26, 55, 75–77].

The HFD has been associated with a significant increase in gut permeability due to disruption of the gut epithelial cell-cell junction integrity, which may be caused by multiple factors, such as components of HFD itself [78] and gut dysbiosis [5, 72, 79, 80]. The dysregulated GM is often associated with decreased short-chain fatty acid (SCFA) production, which is critical in maintaining intestinal epithelial cell-cell junctions through modulating mucin synthesis and regulating TJ protein expression [72, 79, 80]. Furthermore, HFD independently increases gut permeability since the dietary components are reported to directly interact with the signaling pathways modulating the cellular actin-myosin cytoskeleton proteins in the gut epithelial cells, which play a key role in regulating the function of TJ complexes [78]. Several studies have reported that Epac1 regulates endothelial cell-cell junction permeability through modulating a range of molecules that safeguard the endothelial barrier function, including cadherin-mediated cell-cell adhesions, and cytoskeletal reorganization [28, 29]. However, the role of Epac proteins in maintaining the gut epithelial barrier function is currently unclear. We found that Epac2^–/–^ mice had a more significant increase in gut permeability compared to WT mice upon WD feeding, and RD-fed Epac2^–/–^ mice had a significantly lower mRNA expression of TJ protein, ZO-1. Interestingly, it was recently shown that Epac2 activation alleviated colitis in a mouse model by reducing gut permeability and inflammation, and Epac2 protein expression was reduced in the colon of colitis patients [81]. Collectively, these data suggest that Epac2 is critical in maintaining gut barrier function through regulating TJ proteins. While there was no significant difference in the gut permeability between WD-fed Epac1^–/–^ and WT mice, we noted a trend of increased gut permeability in RD-fed Epac1^–/–^ mice. Since Epac1, but not Epac2, protein expression has been detected in different anatomical segments of the small intestine [52], we investigated if WD feeding altered Epac1 protein expression in the jejunum. As shown in Fig. 4e, the Epac1 protein expression was significantly reduced in the jejunum of WD-fed mice compared to RD-fed mice. Therefore, we cannot rule out that reduced Epac1 protein may have a role to play in the gut permeability alterations in WD-fed WT and Epac2^–/–^ mice. Taken together, these data suggest that Epac1 and Epac2 may mediate gut cell-cell permeability during diet-induced obesity.

Deranged signaling in the WAT as consequence of overnutrition is critical in triggering the cascade of events leading to pro-inflammatory, prothrombic, and hyperglycemic state leading to metabolic complications [82]. To investigate if Epac proteins mediate the inflammatory signaling cascade in the WAT, firstly, we examined if Epac1 protein concentration changes in the EWAT upon WD feeding since Epac1 is expressed in the WAT [55, 83]. As shown in Fig. 5a, the Epac1 protein expression was significantly reduced in the EWAT of both WT and Epac2^–/–^ mice fed the WD. Furthermore, WD-fed Epac1^–/–^ mice had significantly increased mRNA levels of pro-inflammatory TNF-α and macrophage infiltration marker, F4/80, in the EWAT compared to the corresponding WT and Epac2^–/–^ mice. However, the pro-inflammatory and macrophage infiltration markers in the EWAT of WD-fed Epac2^–/–^ mice were similar to that of the corresponding WT mice. Altogether, these data suggest that Epac1 is critical in regulating TNF-α signaling and macrophage infiltration, and WD feeding reduces Epac1 protein expression in the WAT, which may lead to increased inflammation. Pro-inflammatory TNF-α has been shown to induce LCN2 [56]; the adipokine LCN2 has been positively correlated with obesity and is reported to mediate insulin resistance in humans [41, 58, 84]. We found that the WD-fed Epac1^–/–^ mice had a significantly higher circulating LCN2 protein than the WD-fed WT mice, suggesting greater systemic inflammation in these mice.

The HFD-associated inflammation is a complex phenomenon with multiple etiologies. The HFD promotes pro-inflammatory state by increasing oxidative stress and endoplasmic reticulum stress [85]. Interestingly, HFD directly stimulates TLR4/ NF-κB signaling, leading to increased local inflammation through upregulation of TNF-α [86], which may contribute to low-grade systemic inflammation associated with insulin resistance. Furthermore, HFD components directly impair the function of TJ complexes resulting in increased gut permeability [78], which allows for mild systemic inflammation and insulin resistance [87]. HFD also causes a decrease in gut peptides, which may also contribute to gut permeability alterations and low-grade inflammation [87]. In the present study, we show that WD reduced Epac1 protein expression in the small intestine and EWAT of WD-fed mice, and Epac1^–/–^ mice exhibited a more significant increase in inflammation and glucose intolerance than in the WT mice after WD feeding. Collectively, these results suggest that Epac1 may mediate the pathogenic mechanisms of HFD. To the best of our knowledge, this is the first time of demonstrating the differential roles of Epac1 and Epac2 in gut permeability changes, inflammation, glucose intolerance, and gut dysbiosis upon WD feeding.

## Conclusions

**Fig. 6:**
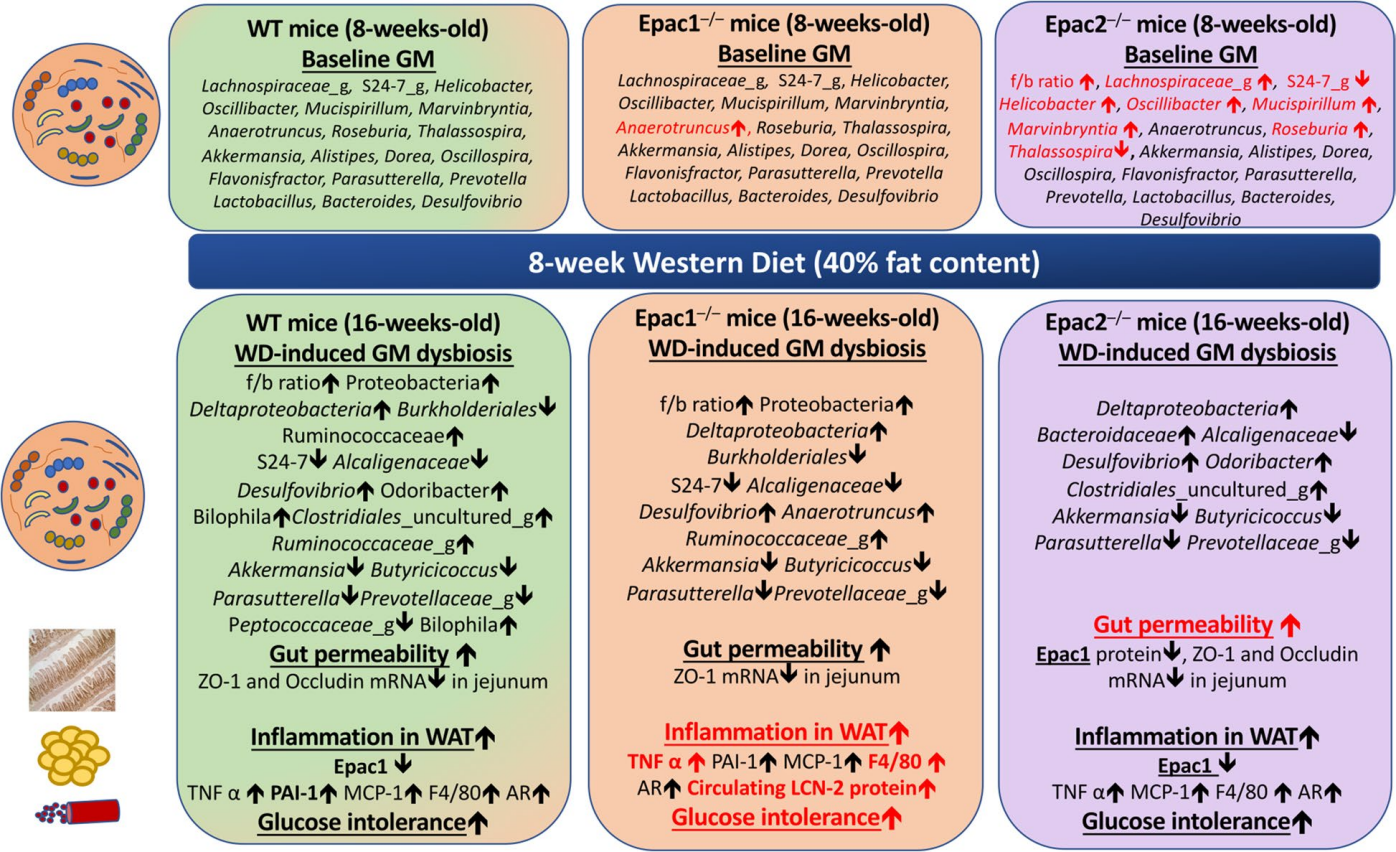
Graphical abstract. This figure highlights the major differences in the baseline GM composition and WD-induced alterations in the GM composition, gut permeability, and inflammation between WT, Epac1^–/–^ and Epac2^–/–^ mice. Statistically significant differences vs. corresponding WT mice are highlighted in red

**Fig. 7:**
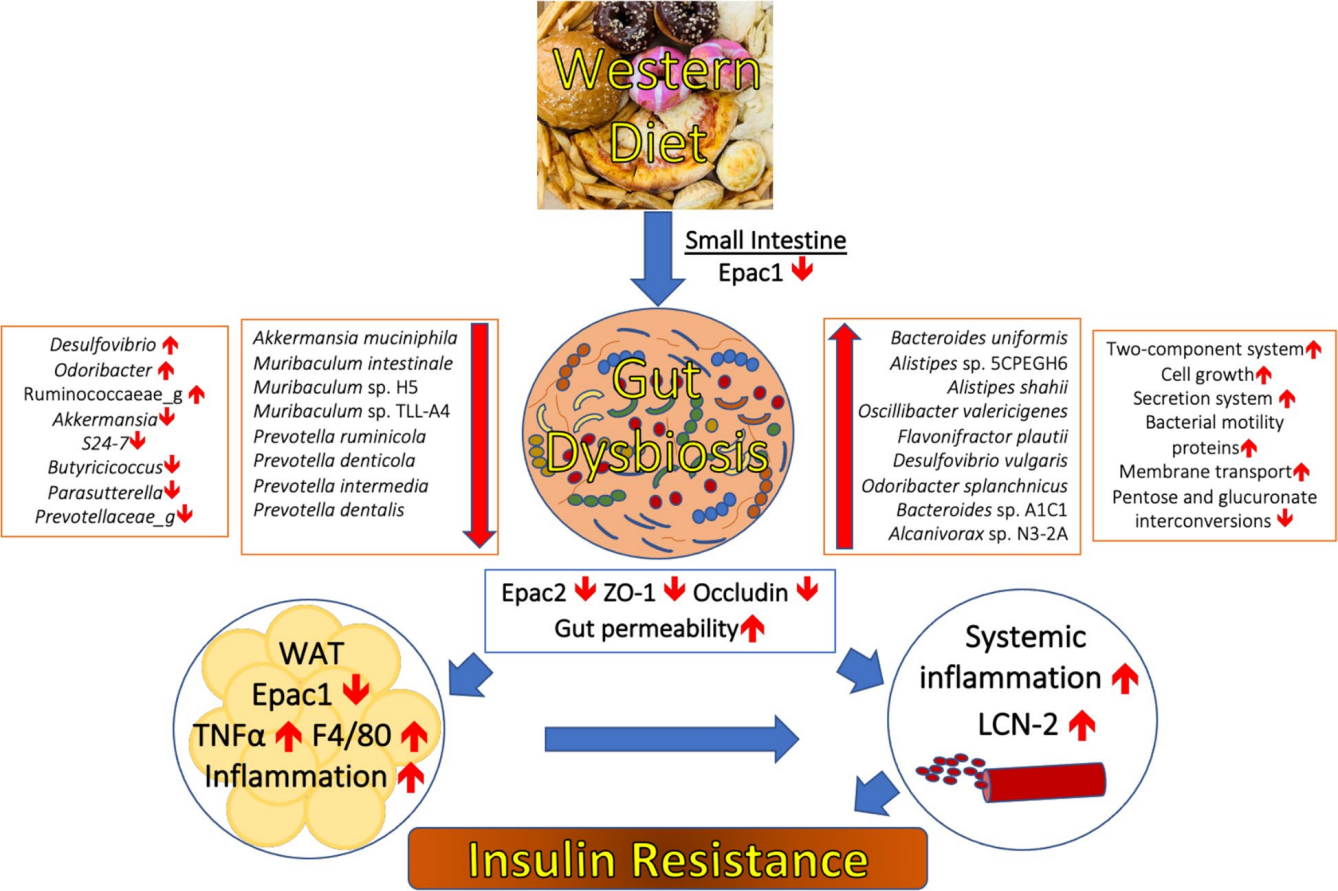
Proposed role of Epac1 in mediating pathophysiology of WD-induced obesity. WD causes a reduction in Epac1 protein in the small intestine and WAT, resulting in increased inflammation and insulin resistance through increased pro-inflammatory TNF-α and F4/80 in the WAT and circulating adipokine LCN2

In the present study, we showed that despite severe dysregulations in the baseline GM and a more significant increase in gut permeability upon WD feeding, the WD-fed Epac2-deficient mice did not exhibit more severe inflammation, glucose intolerance, and gut dysbiosis than WD-fed WT mice (Fig. 6). These data suggest that the role of gut dysbiosis in mediating WD-induced obesity may be context-dependent. Furthermore, we showed that WD reduced Epac1 protein expression in the small intestine and EWAT of WT and Epac2^–/–^ mice, and WD-fed Epac1-deficient mice exhibited a more significant increase in inflammation and glucose intolerance compared to the corresponding WT mice (Fig. 6). In addition, we showed that Epac1 deficiency augmented GM dysbiosis in mice upon WD feeding, albeit modestly. In conclusion, deficiency of Epac1 in mice drove inflammation and glucose intolerance which are the hallmarks of WD-induced obesity (Fig. 7). Further investigations are warranted to explore the therapeutic potential of Epac1 agonists in alleviating WD-induced inflammation, insulin resistance, and gut dysbiosis.

## Supplementary Information

**Additional file 1: Table S1.** RT-qPCR primer sequences for the targeted mouse genes.

**Additional file 2.** R scripts used for bioinformatics analysis.

**Additional file 3: Table S2**. Relative abundance of phyla detected through 16S rRNA gene sequencing using fecal DNA.

**Additional file 4: Table S3.** Relative abundance of classes detected through 16S rRNA gene sequencing using fecal DNA.

**Additional file 5: Table S4.** Relative abundance of orders detected through 16S rRNA gene sequencing using fecal DNA.

**Additional file 6: Table S5.** Relative abundance of families detected through 16S rRNA gene sequencing using fecal DNA.

**Additional file 7: Table S6.** Relative abundance of genera detected through 16S rRNA gene sequencing using fecal DNA.

**Additional file 8: Fig. S1.** Characterization of baseline GM of WT, Epac1^–/–^, and Epac2^–/–^ mice at the class and order levels. **Fig. S2.** Characterization of baseline GM of WT, Epac1^–/–^, and Epac2^–/–^ mice at the family and genus levels. **Fig. S3.** GM alterations at the class and order levels in the WT, Epac1^–/–^ and Epac2^–/–^ mice induced by an 8-week WD. **Fig. S4.** GM alterations at the family level in the WT, Epac1^–/–^ and Epac2^–/–^ mice induced by an 8-week WD. **Fig. S5.** An 8-week WD caused a shift in functional metagenome regardless of genotype. **Fig. S6.** Predominantly altered KEGG pathways due to an 8-week WD or Epac1/ Epac2 deficiency in RD-fed mice. **Fig. S7.** Fed blood sugar levels in the WT, Epac1^–/–^ and Epac2^–/–^ mice after feeding RD or WD for 8 weeks. **Fig. S8.** Gut permeability alterations in WT, Epac1^–/–^ and Epac2^–/–^ mice upon WD feeding compared to their respective RD-fed counterparts. **Fig. S9.** Alterations in the mRNA levels of oxidative stress and adipokines in the liver and EWAT of WT, Epac1^–/–^ and Epac2^–/–^ mice upon WD feeding.

**Additional file 9: Table S7.** Relative abundance of bacterial species determined through shotgun sequencing.

**Additional file 10: Table S8.** Gene coverage of various functional KEGG genes detected through shotgun sequencing.

**Additional file 11: Table S9.** KEGG orthology IDs.

## Data Availability

All processed and analyzed sequencing data related to this study has been included in the manuscript or additional files. The raw shotgun and 16S rRNA gene sequencing data have been deposited in the Sequence Read Archive-National Centre for Biotechnology Information (SRA-NCBI) under the accession number PRJNA812891.

## Abbreviations

Epac: Exchange proteins directly activated by cAMP
GM: Gut microbiota
WD: Western diet
RD: Regular diet
WT: Wild-type
IPGTT: Intraperitoneal glucose tolerance test
HFD: High-fat diet
Da: Dalton
mRNA: Messenger ribonucleic acid
TNF-α: Tumor necrosis factor-alpha
KEGG: Kyoto Encyclopedia of Genes and Genome
EWAT: Epididymal white adipose tissue
LCN2: Lipocalin-2
f/b: *Firmicutes*/ *Bacteroidetes*
cAMP: Cyclic adenosine monophosphate
PKA: Protein kinase A
GEF: Guanine nucleotide exchange factor
RAP1: Ras-related protein 1
TJ: Tight junction
ZO-1: Zona occudens-1
OGTT: Oral glucose tolerance test
FITC: Fluorescein-isothiocyanate
rRNA: Ribosomal ribonucleic acid
RT-qPCR: Real-time quantitative PCR
LPS: Lipopolysaccharide
PAI-1: Plasminogen-activator inhibitor-1
TGF-β: Transforming growth factor beta
MCP-1: Monocyte chemoattractant protein-1
STAMP2: Six transmembrane protein of prostate 2
NAPDHox: Nicotinamide adenine dinucleotide phosphate oxidase
