# Supplementary material for: Deficiency of exchange protein directly activated by cAMP (EPAC)-1 in mice augments glucose intolerance, inflammation, and gut dysbiosis associated with Western diet
Source: Microbiome. 2022 Nov 4;10:187. doi: 10.1186/s40168-022-01366-0 (PMC9635209; doi:10.1186/s40168-022-01366-0)
Supplement: Supplementary file 2 — Additional file 1: Table S1. RT-qPCR primer sequences for the targeted mouse genes. [file 40168_2022_1366_MOESM1_ESM.docx]

| Gene  [Accession ID/ Reference] | Forward | Reverse |
| --- | --- | --- |
| Adiponectin  [GI:87252710] | CACTGTTCCCAATGTACCCA | CCTTCTTGAAGAGGCTCACC |
| LCN2  [GI:34328048] | CAGAAGGCAGCTTTACGATG | TGTGCATATTTCCCAGAGTGA |
| PAI-1 [1] | ACAGCCTTTGTCATCTCAGCC | CCGAACCACAAAGAGAAAGGA |
| F4/80 [1] | TGACAACCAGACGGCTTGTG | GCAGGCGAGGAAAAG ATAGTGT |
| MCP1[1] | GCAGTTAACG CCCCACTCA | CCCAGCCTACTCATTGGGATCA |
| NADPHox [1] | GGTTGGGGCTGAACATTTTTC | TCGACACACAGGAATCAGGAT |
| STAMP2 [1] | GCATCTAGTGTTCCTGACTGGA | TCAAATGCGGAATACCTTGCT |
| RPL19 [1] | GAAGGTCAAAGGGAATGTGTTCA | CCTTGTCTGCCTTCA GCTTGT |
| ZO-1 [2] | TTTTTGACAGGGGGAGTGG | TGCTGCAGAGGTCAAAGTTCAAG |
| Occludin [2] | ATGTCCGGCCGATGCTCTC | TTTGGCTGCTCTTGGGTCTGTAT |
| TNFα [2] | ACGGCATGGATCTCAAAGAC | AGATAGCAAATCGGCTGACG |
| TGF-β1 [3] | TGGAGCAACATGTGGAACTC | CGTCAAAAGACAGCCACTCA |
| GAPDH [2] | CTCATGACCACAGTCCATGC | CACATTGGGGGTAGGAACAC |

**Additional file 1.** **Table S1**. RT-qPCR primer sequences for the targeted mouse genes.

**Additional file 1.** **Table S1**. RT-qPCR primer sequences for the targeted mouse genes. RT-qPCR primer sequences. Abbreviations, LCN2: Lipocalin-2; PAI-1: Plasminogen-activator inhibitor-1; MCP-1: Monocyte chemoattractant protein-1; NAPDHox: Nicotinamide adenine dinucleotide phosphate oxidase; STAMP2: Six transmembrane protein of prostate 2; RPL19: 60S ribosomal protein L19; ZO-1: Zona occudens-1; TNF-α: Tumor necrosis factor-alpha; TGF-β: Transforming growth factor beta; GAPDH: Glyceraldehyde 3-phosphate dehydrogenase.

**References**

1. Cani, P.D., et al., Changes in gut microbiota control metabolic endotoxemia-induced inflammation in high-fat diet-induced obesity and diabetes in mice. Diabetes, 2008. 57(6): p. 1470-81.

2. Li, J., et al., Akkermansia Muciniphila Protects Against Atherosclerosis by Preventing Metabolic Endotoxemia-Induced Inflammation in Apoe-/- Mice. Circulation, 2016. 133(24): p. 2434-46.

3. Zhang, S., et al., Caffeine preferentially protects against oxygen-induced retinopathy. FASEB J, 2017. 31(8): p. 3334-3348.
