# Supplementary material for: Deficiency of exchange protein directly activated by cAMP (EPAC)-1 in mice augments glucose intolerance, inflammation, and gut dysbiosis associated with Western diet
Source: Microbiome. 2022 Nov 4;10:187. doi: 10.1186/s40168-022-01366-0 (PMC9635209; doi:10.1186/s40168-022-01366-0)
Supplement: Supplementary file 3 — Additional file 2. R scripts used for bioinformatics analysis. [file 40168_2022_1366_MOESM2_ESM.pdf]

Epac Manuscript

This document contains all statistical analysis and figure plotting conducted in R for the manuscript.

Load necessary libraries

```
library(vegan)
library(ggpubr)
library(rstatix)
library(mixOmics)
library(pheatmap)
```

Load and pre-process metadata table and OTU count table. Filter features with relative abundance less than 0.1% in less than 5% of the samples. Count tables were normalized into relative abundances for further analysis

```
meta.data <- read.csv("metadata.csv", header = T, row.names = 1)
rownames(meta.data) <- paste0("X",rownames(meta.data))

genus.data <- read.table("otu_table_sorted_L6.txt", header = T)
genus.data <- genus.data[grepl("__[^;]{2,}$",genus.data$Taxon),]
genus.data$Taxon <- gsub(".*;__","",genus.data$Taxon)
genus.data <- genus.data[genus.data$Taxon!="Incertae_Sedis",]
rownames(genus.data) <- genus.data$Taxon; genus.data <- genus.data[,-1]
genus.data <- sweep(genus.data,2,colSums(genus.data),`/`)
filter.feat <- names(which(rowSums(genus.data*100 > 0.1) > .05*ncol(genus.data)))
genus.data <- genus.data[filter.feat,]
genus.data<- sweep(genus.data,2,colSums(genus.data),`/`)
genus.data <- as.data.frame(t(genus.data))

phylum.data <- read.table("otu_table_sorted_L2.txt", header = T)
phylum.data <- phylum.data[grepl(";__",phylum.data$Taxon),]
phylum.data$Taxon <- gsub(".*;__","",phylum.data$Taxon)
rownames(phylum.data) <- phylum.data$Taxon; phylum.data <- phylum.data[,-1]
phylum.data <- sweep(phylum.data,2,colSums(phylum.data),`/`)
filter.feat <- names(which(rowSums(phylum.data*100 > 0.1) > .05*ncol(phylum.data)))
phylum.data <- phylum.data[filter.feat,]
phylum.data<- sweep(phylum.data,2,colSums(phylum.data),`/`)
phylum.data <- as.data.frame(t(phylum.data))
```

Figure 1 (A) Phylum relative abundance stacked barplot

```
#Specify data subset
dat <- phylum.data[meta.data$Age=="8w",]
dat <- dat[,colSums(dat)!=0]
met <- meta.data[rownames(dat),]

#Data wrangling for plotting
max_feat <- 10
if(ncol(dat)>=max_feat){
  other_col <- colnames(dat)[which(!colnames(dat) %in% names(sort(colSums(dat),decreasing
↪   = T)[1:max_feat-1]))]
  dat$Other <- rowSums(dat[,other_col])
```

```
  dat <- dat[,-which(colnames(dat) %in% other_col)]
}
dat.m <- reshape2::melt(as.matrix(dat))
colnames(dat.m) <- c("Patient","Feat","Abund")
dat.m$Genotype <- as.factor(met[match(dat.m$Patient,rownames(met)),"Genotype"])
dat.m$Genotype <-
↪  ifelse(dat.m$Genotype=="WT","WT",ifelse(dat.m$Genotype=="Epac1-KO","Epac1-/-","Epac2-/-"))
dat.m <- transform(dat.m,Genotype = factor(Genotype,levels
↪  =c("WT","Epac1-/-","Epac2-/-")))
ggplot(dat.m, aes(fill=Feat, y=Abund, x=Genotype)) + geom_bar(position="fill",
↪  stat="identity") + ylab("Relative Abundance") +
  scale_fill_brewer(palette="Paired") +
  theme(legend.title = element_blank(), legend.text = element_text(size = 10, face =
  ↪  "bold.italic"), axis.text = element_text(size = 10, face = "bold"),
        axis.title = element_text(size = 10, face = "bold"))
```

Figure 1 (C) PLS-DA

```
#Specify data subset
dat <- genus.data[meta.data$Age=="8w",]
dat <- dat[,colSums(dat)!=0]
met <- meta.data[rownames(dat),]

#Plotting PLS-DA
lab <-
↪  ifelse(met$Genotype=="WT","WT",ifelse(met$Genotype=="Epac1-KO","Epac1-/-","Epac2-/-"))
plsda <- plsda(dat, lab)
plotIndiv(plsda, ellipse = TRUE, legend = T, title = "Genotype", point.lwd = 1.5,  cex =
↪  3, pch = 16,
          size.xlabel = rel(1.5), size.ylabel = rel(1.5), col = c("red","blue","black"),
          size.legend = 15, legend.title = "",size.axis =15)
plotLoadings(plsda, contrib = 'max', method = 'mean', comp=1, ndisplay = 20, legend.color
↪  = c("red","blue","black"),
             size.name = 0.8, legend = FALSE, layout = c(1,2), title = "Comp 1")
plotLoadings(plsda, contrib = 'max', method = 'mean', comp=2, ndisplay = 20, legend.color
↪  = c("red","blue","black"),
             size.name = 0.8, legend = FALSE, title = "Comp 2")
```

Figure 2 (A) Phylum relative abundance stacked barplot

```
#Specific data subset
dat <- phylum.data[meta.data$Age=="16w",]
dat <- dat[,colSums(dat)!=0]
met <- meta.data[rownames(dat),]

#Outlier mice are X40 and X71
dat <- dat[rownames(dat)!="X40"&rownames(dat)!="X71",]
met <- met[rownames(dat),]

#Data wrangling for plotting
max_feat <- 10
if(ncol(dat)>=max_feat){
```

```
  other_col <- colnames(dat)[which(!colnames(dat) %in% names(sort(colSums(dat),decreasing
↪   = T)[1:max_feat-1]))]
  dat$Other <- rowSums(dat[,other_col])
  dat <- dat[,-which(colnames(dat) %in% other_col)]
}
dat.m <- reshape2::melt(as.matrix(dat))
colnames(dat.m) <- c("Patient","Feat","Abund")
dat.m$Genotype <- as.factor(met[match(dat.m$Patient,rownames(met)),"Genotype"])
dat.m$Diet <- as.factor(met[match(dat.m$Patient,rownames(met)),"Diet"])
dat.m$Genotype <-
↪   ifelse(dat.m$Genotype=="WT","WT",ifelse(dat.m$Genotype=="Epac1-KO","Epac1-/-","Epac2-/-"))
dat.m <- transform(dat.m,Genotype = factor(Genotype,levels
↪   =c("WT","Epac1-/-","Epac2-/-")))
ggplot(dat.m, aes(fill=Feat, y=Abund, x=Diet)) + geom_bar(position="fill",
↪   stat="identity") + ylab("Relative Abundance") +
  scale_fill_brewer(palette="Paired") + facet_wrap(~Genotype) +
  theme(legend.title = element_blank(), legend.text = element_text(size = 10, face =
  ↪   "bold.italic"), axis.text = element_text(size = 10, face = "bold"),
        strip.text = element_text(size = 10, face = "bold"), axis.title =
        ↪   element_text(size = 10, face = "bold"))
```

PLS plots including: Figure 2 (C-D) Figure 3 (A)

```
#Specify data subset
dat <- genus.data[meta.data$Age=="16w",]
dat <- dat[,colSums(dat)!=0]
met <- meta.data[rownames(dat),]
met$Genotype <-
↪   ifelse(met$Genotype=="WT","WT",ifelse(met$Genotype=="Epac1-KO","Epac1-/-","Epac2-/-"))

#Outlier mice are X40 and X71
dat <- dat[rownames(dat)!="X40"&rownames(dat)!="X71",]
met <- met[rownames(dat),]

#Group by Genotype
WT.subset <- dat[met$Genotype=="WT",]
epac1.subset <- dat[met$Genotype=="Epac1-/-",]
epac2.subset <- dat[met$Genotype=="Epac2-/-",]
plsda <- plsda(WT.subset, met[met$Genotype=="WT","Diet"])
plotIndiv(plsda, ellipse = T, legend = T, title = "WT", point.lwd = 1.5,  cex = 3, pch =
↪   16,
          size.xlabel = rel(1.5), size.ylabel = rel(1.5),
          size.legend = 15, legend.title = "",size.axis =15)
plotLoadings(plsda, contrib = 'max', method = 'mean', comp=1, ndisplay = 20, title =
↪   "WT\nComp 1",
             size.title = 1.5,size.name = 0.7, legend = FALSE, layout = c(1,2))
plotLoadings(plsda, contrib = 'max', method = 'mean', comp=2, ndisplay = 20, title =
↪   "WT\nComp 2",
             size.title = 1.5,size.name = 0.7, legend = FALSE)

plsda <- plsda(epac1.subset, met[met$Genotype=="Epac1-/-","Diet"])
plotIndiv(plsda, ellipse = T, legend = T, title = "Epac1-/-", point.lwd = 1.5,  cex = 3,
↪   pch = 16,
```

```
         size.xlabel = rel(1.5), size.ylabel = rel(1.5),
         size.legend = 15, legend.title = "",size.axis =15)
plotLoadings(plsda, contrib = 'max', method = 'mean', comp=1, ndisplay = 20, title =
↪  "Epac1-/-\nComp 1",
            size.title = 1.5,size.name = 0.7, legend = FALSE, layout = c(1,2))
plotLoadings(plsda, contrib = 'max', method = 'mean', comp=2, ndisplay = 20, title =
↪  "Epac1-/-\nComp 2",
            size.title = 1.5,size.name = 0.7, legend = FALSE)

plsda <- plsda(epac2.subset, met[met$Genotype=="Epac2-/-","Diet"])
plotIndiv(plsda, ellipse = T, legend = T, title = "Epac2-/-", point.lwd = 1.5,  cex = 3,
↪  pch = 16,
         size.xlabel = rel(1.5), size.ylabel = rel(1.5),
         size.legend = 15, legend.title = "",size.axis =15)
plotLoadings(plsda, contrib = 'max', method = 'mean', comp=1, ndisplay = 20, title =
↪  "Epac2-/-\nComp 1",
            size.title = 1.5,size.name = 0.7, legend = FALSE, layout = c(1,2))
plotLoadings(plsda, contrib = 'max', method = 'mean', comp=2, ndisplay = 20, title =
↪  "Epac2-/-\nComp 2",
            size.title = 1.5,size.name = 0.7, legend = FALSE)

#Group by Diet
RD.subset <- dat[met$Diet=="RD",]
WD.subset <- dat[met$Diet=="WD",]
plsda <- plsda(RD.subset, met[met$Diet=="RD","Genotype"])
plotIndiv(plsda, ellipse = T, legend = T, title = "RD", point.lwd = 1.5,  cex = 3, pch =
↪  16,
         size.xlabel = rel(1.5), size.ylabel = rel(1.5), col = c("red","blue","black"),
         size.legend = 15, legend.title = "",size.axis =15)
plotLoadings(plsda, contrib = 'max', method = 'mean', comp=1, ndisplay = 20, legend.color
↪  = c("red","blue","black"),
            size.title = 1.5,size.name = 0.7, legend = FALSE, title = "RD\nComp 1",
             ↪  layout = c(1,2))
plotLoadings(plsda, contrib = 'max', method = 'mean', comp=2, ndisplay = 20, legend.color
↪  = c("red","blue","black"),
            size.title = 1.5,size.name = 0.7, legend = FALSE, title = "RD\nComp 2")

plsda <- plsda(WD.subset, met[met$Diet=="WD","Genotype"])
plotIndiv(plsda, ellipse = T, legend = T, title = "WD", point.lwd = 1.5,  cex = 3, pch =
↪  16,
         size.xlabel = rel(1.5), size.ylabel = rel(1.5), col = c("red","blue","black"),
         size.legend = 15, legend.title = "",size.axis =15)
plotLoadings(plsda, contrib = 'max', method = 'mean', comp=1, ndisplay = 20, legend.color
↪  = c("red","blue","black"),
            size.title = 1.5,size.name = 0.7, legend = FALSE, title = "WD\nComp 1",
             ↪  layout = c(1,2))
plotLoadings(plsda, contrib = 'max', method = 'mean', comp=2, ndisplay = 20, legend.color
↪  = c("red","blue","black"),
            size.title = 1.5,size.name = 0.7, legend = FALSE, title = "WD\nComp 2")
```

Supplementary figure 2 (E) Shannon diversity plot

```
#Specific data subset
dat <- genus.data[meta.data$Age=="8w",]
dat <- dat[,colSums(dat)!=0]
met <- meta.data[rownames(dat),]

#Calculate shannon diversity using vegan package
shannon.diversity <- vegan::diversity(dat, index = "shannon")
merged.dat <- data.frame(met, shannon.diversity)
merged.dat$Genotype <-
↪ ifelse(merged.dat$Genotype=="WT","WT",ifelse(merged.dat$Genotype=="Epac1-KO","Epac1-/-","Epac2-/-"))
merged.dat <- merged.dat[merged.dat$shannon.diversity>2,] # Remove outlier | Simple fix
stat.test <- merged.dat %>% pairwise_wilcox_test(shannon.diversity ~ Genotype,
↪ p.adjust.method = "BH") %>% add_y_position()
ggboxplot(merged.dat,"Genotype", "shannon.diversity", lwd = 1) +
  stat_pvalue_manual(stat.test[-1,], label = "p.adj.signif", tip.length = 0.01
  ↪ ,step.increase = 0.05, bracket.size = 1, label.size = 5, bracket.nudge.y = 0.01) +
  theme(axis.title = element_text(size = 16, face="bold"), axis.text = element_text(size
  ↪ = 16, face="bold"))
```

Supplementary figure 2 (F) Heatmap of top 20 genera of baseline mice

```
#Specific data subset
dat <- genus.data[meta.data$Age=="8w",]
dat <- dat[,colSums(dat)!=0]
met <- meta.data[rownames(dat),]

topn <- rev(names(sort(colSums(dat),decreasing = T)[1:20]))
merged.dat <- cbind(dat,met)
colnames(merged.dat) <- make.names(colnames(merged.dat))
sig.feat <- data.frame(feat = character(), group1 = character(), group2 = character(),
↪ p.adj = character(), significance = character())
for(bac in topn){
  f <- formula(paste0(bac, " ~ Genotype"))
  stat.test <- merged.dat %>% wilcox_test(f, comparisons = list(c("WT", "Epac1-KO"),
↪ c("WT", "Epac2-KO")))
  #sig <- stat.test[grepl("\\*",stat.test$p.adj.signif),]
  sig.feat <- rbind(sig.feat,stat.test[,c(1:3,8:9)])
}

melt.dat <- reshape2::melt(as.matrix(dat[,unique(sig.feat$.y.)]))
colnames(melt.dat) <- c("ID","Feat","Abundance")
melt.dat$Genotype <- met[match(melt.dat$ID,rownames(met)),"Genotype"]
melt.dat$Diet <- met[match(melt.dat$ID,rownames(met)),"Diet"]
melt.dat <- aggregate(Abundance ~ Feat + Genotype + Diet, melt.dat, FUN = mean)
melt.dat$LogAbundance <- log(melt.dat$Abundance+0.000001)
melt.dat <- merge(melt.dat, sig.feat, by.x=c('Feat','Genotype'), by.y=c('.y.',"group1"),
↪ all.x = T)
melt.dat$Genotype <-
↪ ifelse(melt.dat$Genotype=="WT","WT",ifelse(melt.dat$Genotype=="Epac1-KO","Epac1-/-","Epac2-/-"))
melt.dat <- transform(melt.dat,Genotype = factor(Genotype,levels
↪ =c("WT","Epac1-/-","Epac2-/-")))
melt.dat[melt.dat$Genotype=="WT","p.adj.signif"] <- NA
melt.dat[which(melt.dat$p.adj.signif=="ns"),"p.adj.signif"] <- NA
```

```
#With statitics
ggplot(melt.dat, aes(Genotype, Feat)) + geom_tile(aes(fill = LogAbundance)) +
↪  geom_text(aes(label=p.adj.signif), size = 6) +
  scale_fill_gradientn(colours = rainbow(2)) +
  theme(panel.grid.major = element_blank(), panel.grid.minor = element_blank()) +
  ↪  ylab("") +
  theme(axis.title = element_text(size = 12, face="bold"), axis.text = element_text(size
  ↪  = 12, face="bold"),
        axis.text.y = element_text(face="bold.italic"),strip.text = element_text(size=12,
        ↪  face="bold")) +
  theme(legend.title = element_text(size = 12, face="bold"), legend.text =
  ↪  element_text(size = 10, face="bold"))
#Without statistics
ggplot(melt.dat, aes(Genotype, Feat)) + geom_tile(aes(fill = LogAbundance)) +
  scale_fill_gradientn(colours = rainbow(2)) +
  theme(panel.grid.major = element_blank(), panel.grid.minor = element_blank()) +
  ↪  ylab("") +
  theme(axis.title = element_text(size = 12, face="bold"), axis.text = element_text(size
  ↪  = 12, face="bold"),
        axis.text.y = element_text(face="bold.italic"),strip.text = element_text(size=12,
        ↪  face="bold")) +
  theme(legend.title = element_text(size = 12, face="bold"), legend.text =
  ↪  element_text(size = 10, face="bold"))
```

Figure 3 (C) Metagenome species-level hierarchical clustering heatmap

```
#Create metadata table for pool metagenome samples
meta.data <- data.frame(c("control","group1","group2","group3","group4","group5"),
                        Genotype = c("WT","WT","Epac1-/-", "Epac1-/-", "Epac2-/-",
                        ↪  "Epac2-/-"),
                        Diet = rep(c("RD","WD"),3), row.names = 1)

#Load and pre-process metagenome read count data
species.data <- read.table("shotgun_species.csv", sep = ",", header = T, row.names = 1)
species.data <- sweep(species.data,2,colSums(species.data),'/')
filt.feat.more <- names(which(rowSums(species.data*100 > 1) > .05*ncol(species.data)))
species.data.filt.more <- species.data[filt.feat.more,]

#Cluster and plot heatmap using pheatmap library
pheatmap(species.data.filt.more, annotation_col=meta.data,
         annotation_names_row=F,
         annotation_names_col=F,
         fontsize_row=15,
         fontsize = 15,
         show_colnames = F, cellwidth = 50, cellheight=16)
```

Supplementary figure 6 Functional metagenome heatmap

```
gene.data <- read.table("gene_coverage_comparison.csv", sep = ",", header = T, row.names
↪  = 1)
ko.table <- read.delim("full_KO_table.csv", sep = ",", header = T)
```

```
ko.table$KO.Desc.D <- gsub("^ *", "", ko.table$KO.Desc.D)

gene.data <- gene.data[rowSums(gene.data)>100,]

bio.dat <- data.frame(gene.data,bio.process =
↪  ko.table[match(rownames(gene.data),ko.table$KO.ID.D),"KO.Desc.C"])
bio.dat <- aggregate(.~bio.process,bio.dat, FUN = sum)
rownames(bio.dat) <- bio.dat$bio.process; bio.dat <- bio.dat[,-1]
bio.dat <- bio.dat[rowSums(bio.dat)>median(rowSums(bio.dat)),]

cell.dat <- data.frame(gene.data,cell.dat =
↪  ko.table[match(rownames(gene.data),ko.table$KO.ID.D),"KO.Desc.B"])
cell.dat <- aggregate(.~cell.dat,cell.dat, FUN = sum)
rownames(cell.dat) <- cell.dat$cell.dat; cell.dat <- cell.dat[,-1]

#Select features
bio.feat <- c("Riboflavin metabolism [PATH:ko00740]","RNA degradation [PATH:ko03018]",
                                "Ubiquinone and other terpenoid-quinone biosynthesis
                                ↪  [PATH:ko00130]",
                                "Pentose and glucuronate interconversions
                                ↪  [PATH:ko00040]",
                                "Lipopolysaccharide biosynthesis [PATH:ko00540]",
                                ↪  "Bacterial motility proteins [BR:ko02035]",
                                "Methane metabolism [PATH:ko00680]","Bacterial secretion
                                ↪  system [PATH:ko03070]",
                                "Secretion system [BR:ko02044]", "Two-component system
                                ↪  [PATH:ko02020]",
                                "Inorganic ion transport and metabolism","Cell
                                ↪  growth","Sulfur metabolism [PATH:ko00920]",
                                "Butanoate metabolism [PATH:ko00650]", "Propanoate
                                ↪  metabolism [PATH:ko00640]")
cellular.feat <- c("Biosynthesis of other secondary metabolites", "Glycan biosynthesis
↪  and metabolism", "Cell motility",
                   "Signal transduction","Membrane transport", "Nucleotide metabolism",
                   ↪  "Carbohydrate metabolism",
                   "Amino acid metabolism","Lipid metabolism","Energy metabolism","Enzyme
                   ↪  families")
dat <- bio.dat[bio.feat,]
dat2 <- cell.dat[cellular.feat,]
info.table <- info.table[,-1, drop = F]
pheatmap(dat, annotation_col=meta.data,
         annotation_names_row=F,
         annotation_names_col=F,
         show_rownames = T,
         cluster_rows = F,
         cluster_cols = F,
         fontsize_row=15,
         fontsize = 15)
pheatmap(dat2, annotation_col=meta.data,
         annotation_names_row=F,
         annotation_names_col=F,
         show_rownames = T,
         cluster_rows = F,
```

```
         cluster_cols = F,
         fontsize_row=15,
         fontsize = 15)
```
