# Supplementary material for: Deficiency of exchange protein directly activated by cAMP (EPAC)-1 in mice augments glucose intolerance, inflammation, and gut dysbiosis associated with Western diet
Source: Microbiome. 2022 Nov 4;10:187. doi: 10.1186/s40168-022-01366-0 (PMC9635209; doi:10.1186/s40168-022-01366-0)
Supplement: Supplementary file 9 — Additional file 8: Fig. S1. Characterization of baseline GM of WT, Epac1–/–, and Epac2–/– mice at the class and order levels. Fig. S2. Characterization of baseline GM of WT, Epac1–/–, and Epac2–/– mice at the family and genus levels. Fig. S3. GM alterations at the class and order levels in the WT, Epac1–/– and Epac2–/– mice induced by an 8-week WD. Fig. S4. GM alterations at the family level in the WT, Epac1–/– and Epac2–/– mice induced by an 8-week WD. Fig. S5. An 8-week WD caused a shift in functional metagenome regardless of genotype. Fig. S6. Predominantly altered KEGG pathways due to an 8-week WD or Epac1/ Epac2 deficiency in RD-fed mice. Fig. S7. Fed blood sugar levels in the WT, Epac1–/– and Epac2–/– mice after feeding RD or WD for 8 weeks. Fig. S8. Gut permeability alterations in WT, Epac1–/– and Epac2–/– mice upon WD feeding compared to their respective RD-fed counterparts. Fig. S9. Alterations in the mRNA levels of oxidative stress and adipokines in the liver and EWAT of WT, Epac1–/– and Epac2–/– mice upon WD feeding. [file 40168_2022_1366_MOESM8_ESM.pdf]

Fig. S1

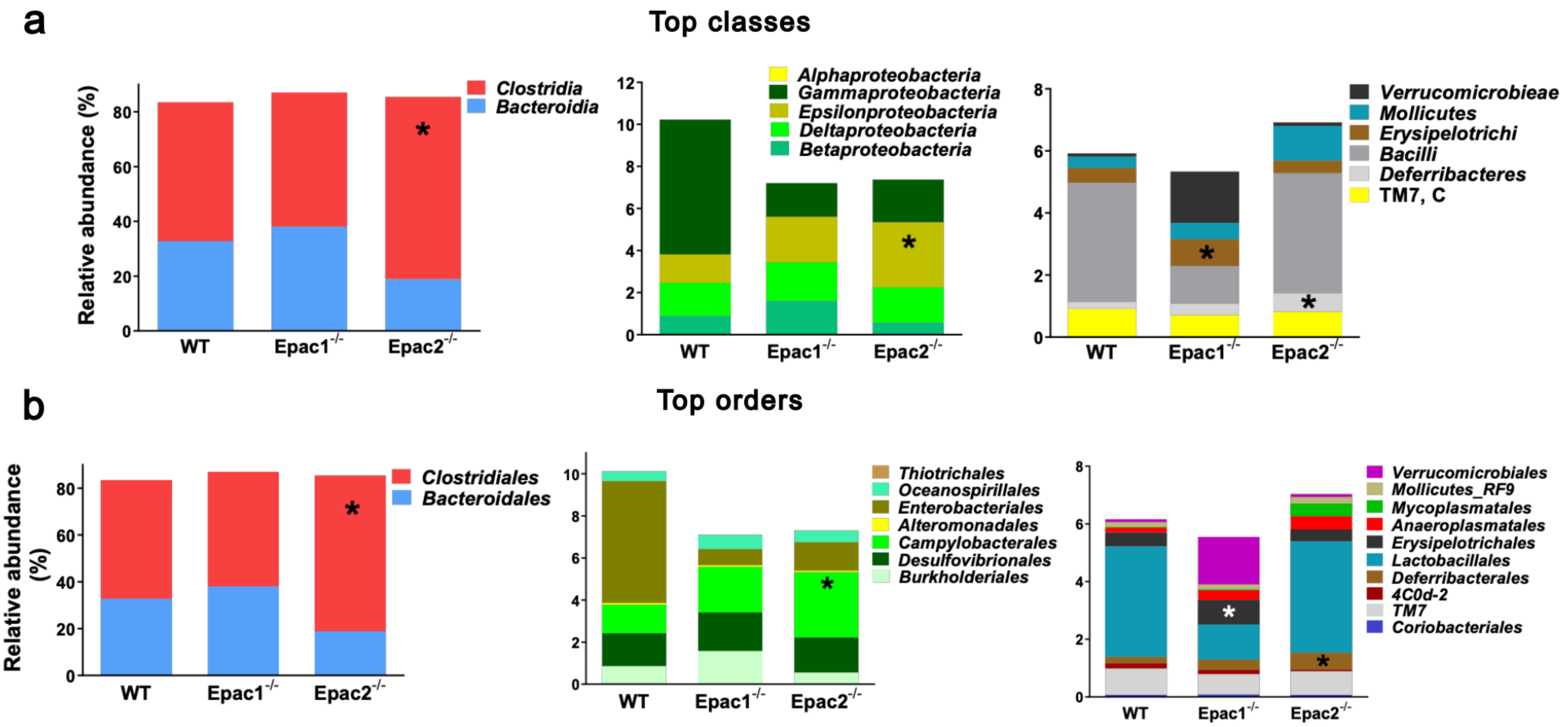

Fig. S1. Characterization of baseline GM of WT, Epac1$^{-/-}$, and Epac2$^{-/-}$ mice at the class and order levels. All data are from 8-week-old mice fed the RD (n=8/genotype)**.** The bacterial taxa abundance was determined by16S rRNA gene sequencing using fecal DNA. Stacked barplots showing the relative abundance of predominant bacterial (**a**) classes and (**b**) orders. The barplots are segregated into 3 separate panels for the sake of clarity. All data are presented as mean and analyzed by one-way ANOVA with Tukey's post hoc test, *P<0.05 vs. WT mice.

Fig. S2

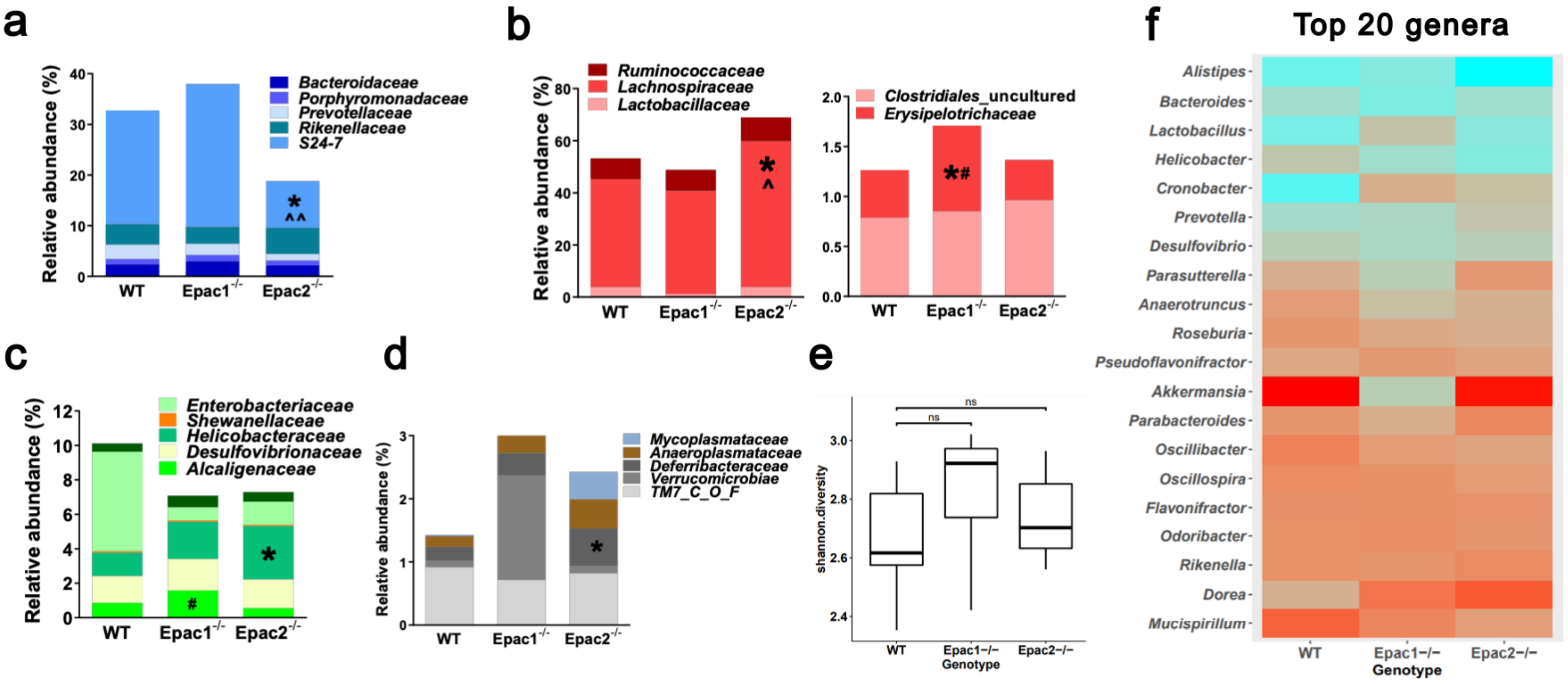

Fig. S2. Characterization of baseline GM of WT, Epac1$^{-/-}$, and Epac2$^{-/-}$ mice at the family and genus levels. All data are from 8-week-old mice fed the RD (n=8/genotype)**.** The bacterial taxa abundance was determined by16S rRNA gene sequencing using fecal DNA. Stacked barplots showing the relative abundance of predominant bacterial families in the phylum (**a**) *Bacteroidetes*; (**b**) *Firmicutes*; (**c**) *Proteobacteria*; (**d**) Others. Data are presented as mean. (**e**) Shannon's diversity index based on genera abundances; data were analyzed by Wilcoxon rank sum test with Benjamini-Hochberg multiple hypothesis testing adjustment, non significant (ns)**.** (**f**) Heatmap showing the relative abundance of top 20 genera within each genotype. Scale of the heatmap is represented as the log relative abundance with the addition of a small pseudo value to avoid log zero. **(Fig. a-d,f)** Data were analyzed by one-way ANOVA with Tukey's post hoc test, *P<0.05 vs. WT, ^P<0.05, ^^P<0.01 vs. Epac1$^{-/-}$ and #P<0.05 vs. Epac2$^{-/-}$ mice.

Fig. S3

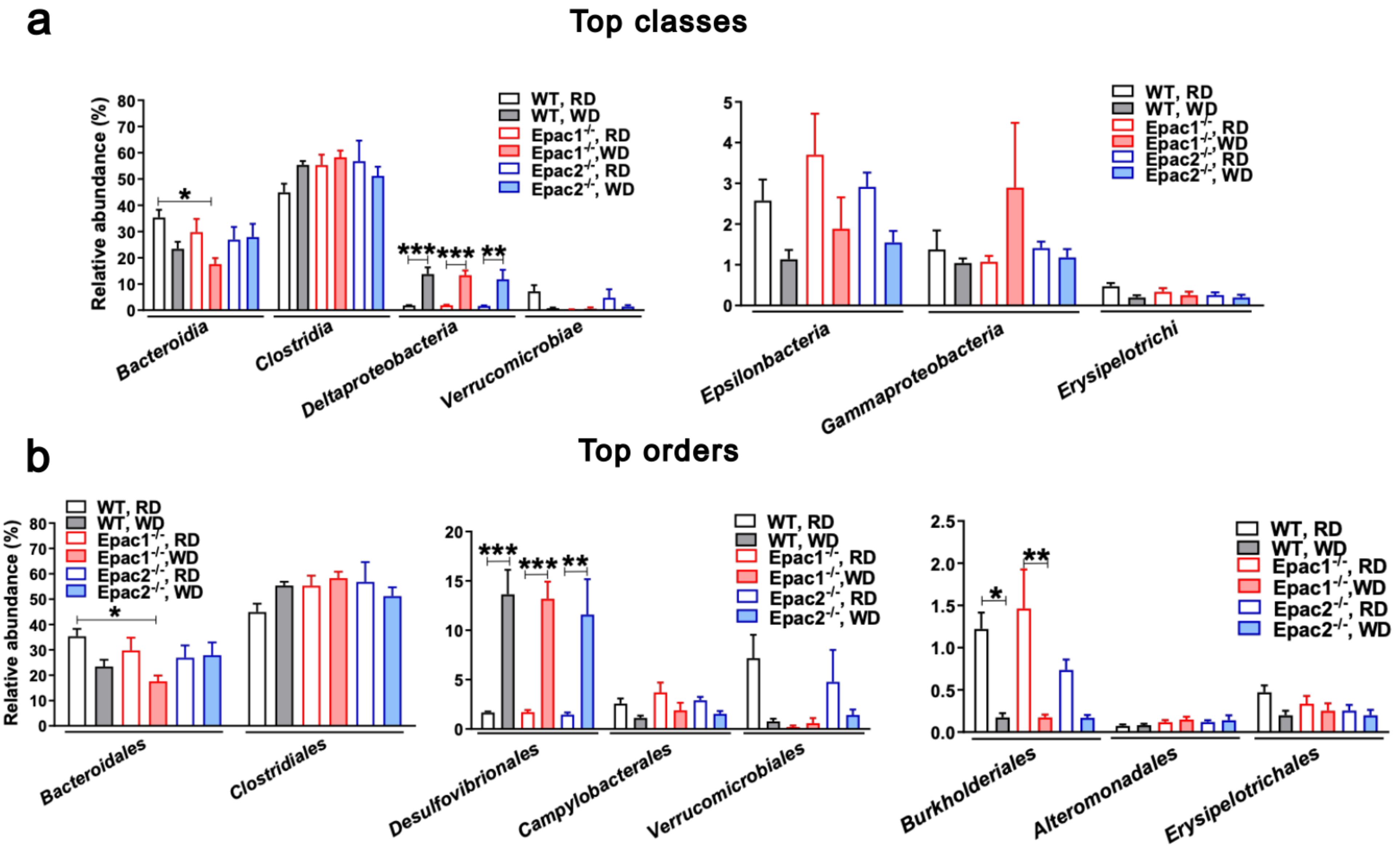

Fig. S3. GM alterations at the class and order levels in the WT, Epac1$^{-/-}$ and Epac2$^{-/-}$ mice induced by an 8-week WD. All data are from 16-week-old mice (8-week-old + RD or WD for 8 weeks). Relative abundance of predominant bacterial (**a**) classes; (**b**) orders. The bar plots are segregated into separate panels for the sake of clarity. Statistical significance *P<0.05, **P<0.01, ***P<0.001, was assessed by two-way ANOVA with Tukey's test (n=7-8/genotype). All data are presented as mean ± SEM.

Fig. S4

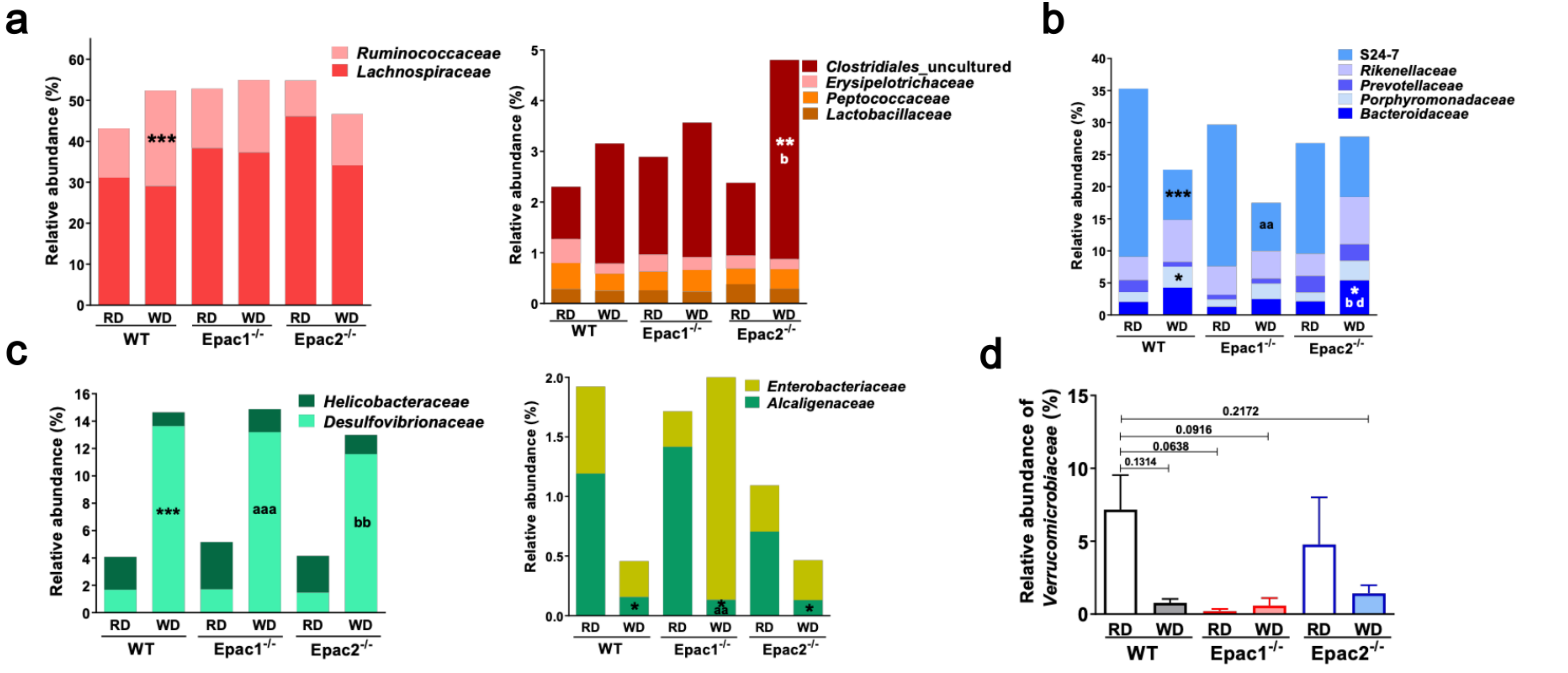

Fig S4. GM alterations at the family level in the WT, Epac1$^{-/-}$ and Epac2$^{-/-}$ mice induced by an 8-week WD. All data are from 16-week-old mice (8-week-old + RD or WD for 8 weeks). The relative abundance of predominant bacterial families detected and significant differences in the abundance among the genotypes and diets are shown in the stacked bar plots. Predominant families in the phylum (**a**) *Firmicutes*; (**b**) *Bacteroidetes*; (**c**) *Proteobacteria*. The stacked bar plots a and c are segregated into separate panels for the sake of clarity. (**d**) *Verrucomicrobiaceae*. Statistical significance *P<0.05, **P<0.01 and ***P<0.001, vs. RD-fed WT mice; ^{a}P<0.05, ^{aa}P<0.01 and ^{aaa}P<0.001 vs. RD-fed Epac1$^{-/-}$, ^{b}P<0.05 and ^{bb}P<0.01 vs. RD-fed Epac2, and ^{d}P<0.05 vs. WD-fed Epac1 was mice was assessed by two-way ANOVA with Tukey's test (n=7-8/genotype). The data are presented as mean (a-c) and mean ± SEM (d).

Fig. S5

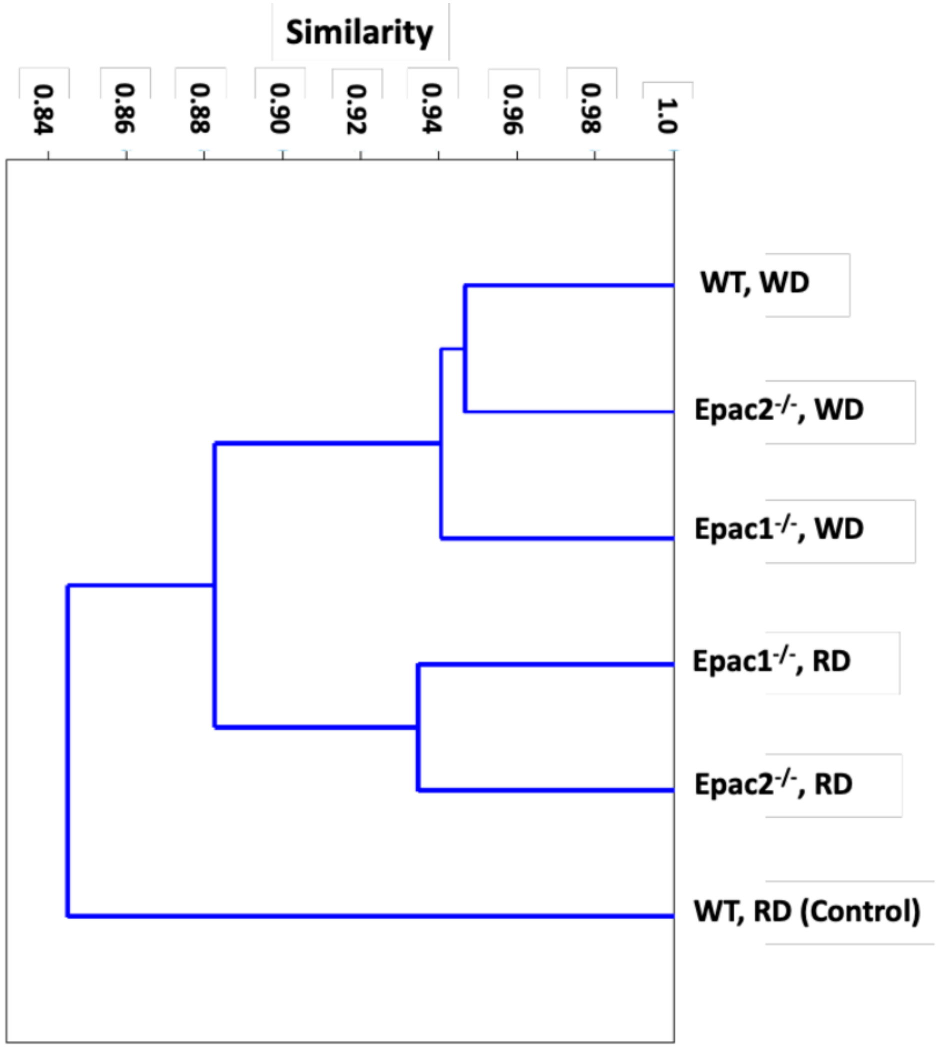

Fig S5. An 8-week WD caused a shift in functional metagenome regardless of genotype. Shotgun sequencing using fecal DNA from composite samples by pooling DNA from 6 mice from 3 genotypes X 2 diets. All mice were 16-week-old (8-week-old + RD or WD for 8 weeks). A PAST cluster analysis showing the similarity between RD and WD-fed groups based on the gene coverage values.

Fig. S6

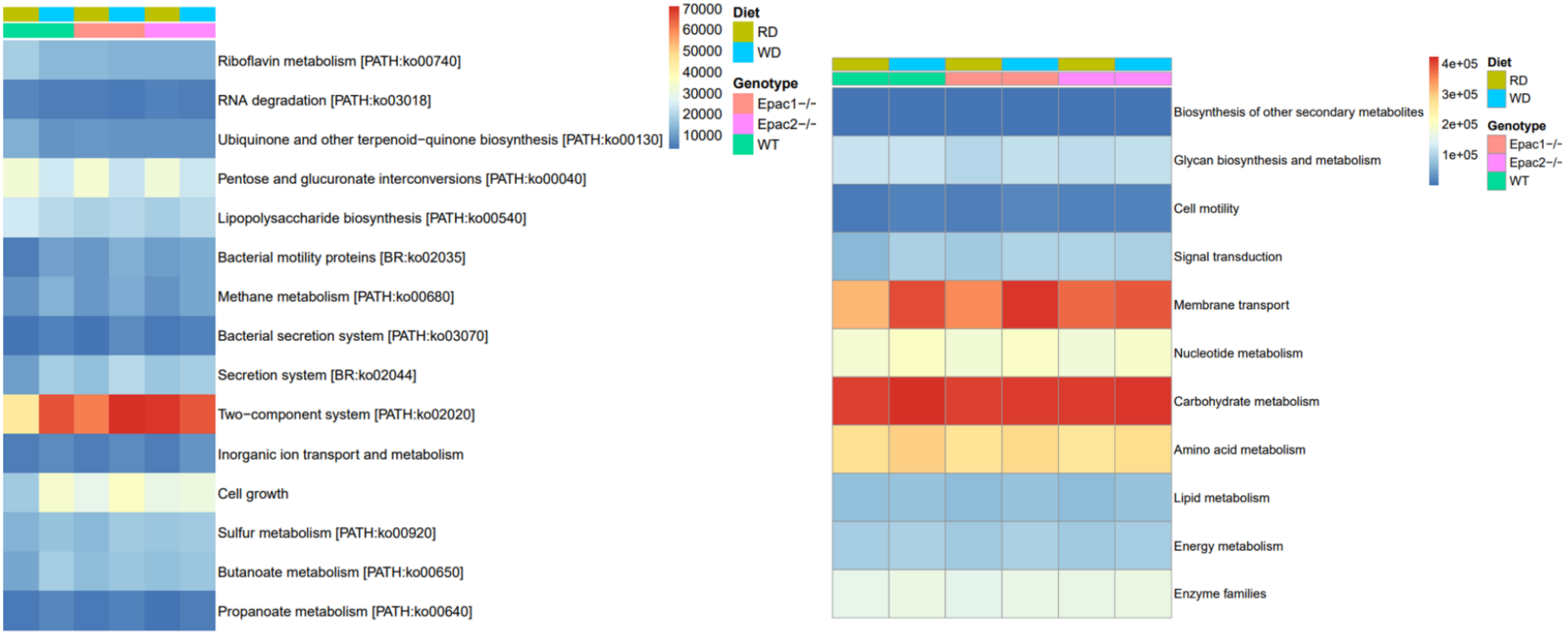

Fig S6. Predominantly altered KEGG pathways due to an 8-week WD or Epac1/ Epac2 deficiency in RD-fed mice. Shotgun sequencing using fecal DNA from composite samples by pooling DNA from 6 mice from 3 genotypes X 2 diets. Heatmaps showing alterations in the abundance of functional genes in KEGG pathways due to WD feeding or Epac1 or Epac2 deficiency. The colored tiles represent the gene coverage and the scale bar is alongside the heatmap.

Fig. S7

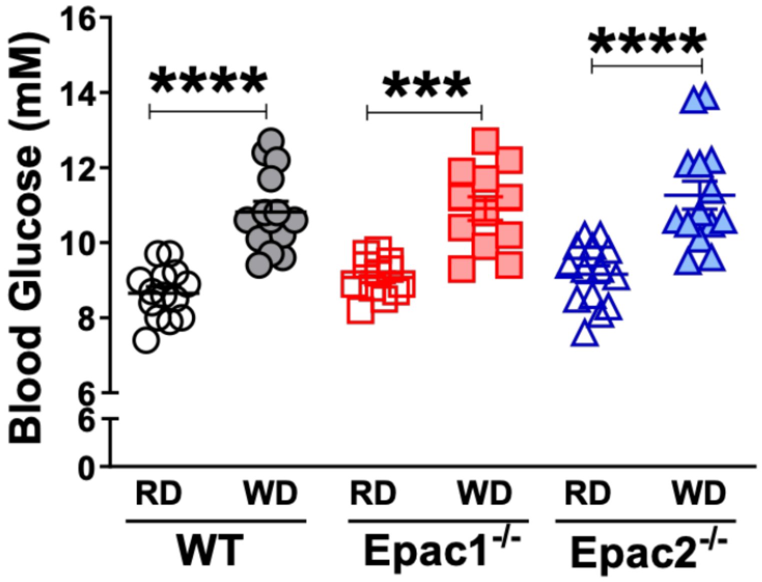

Fig S7. Fed blood sugar levels in the WT, Epac1$^{-/-}$ and Epac2$^{-/-}$ mice after feeding RD or WD for 8 weeks. These data are from 16-week-old mice (8-week-old + RD or WD for 8 weeks). Statistical significance ***P<0.001, ****P<0.0001 was assessed by two-way ANOVA with Tukey's test (n=12-14/genotype/diet). Data are presented as mean ± SEM.

Fig. S8

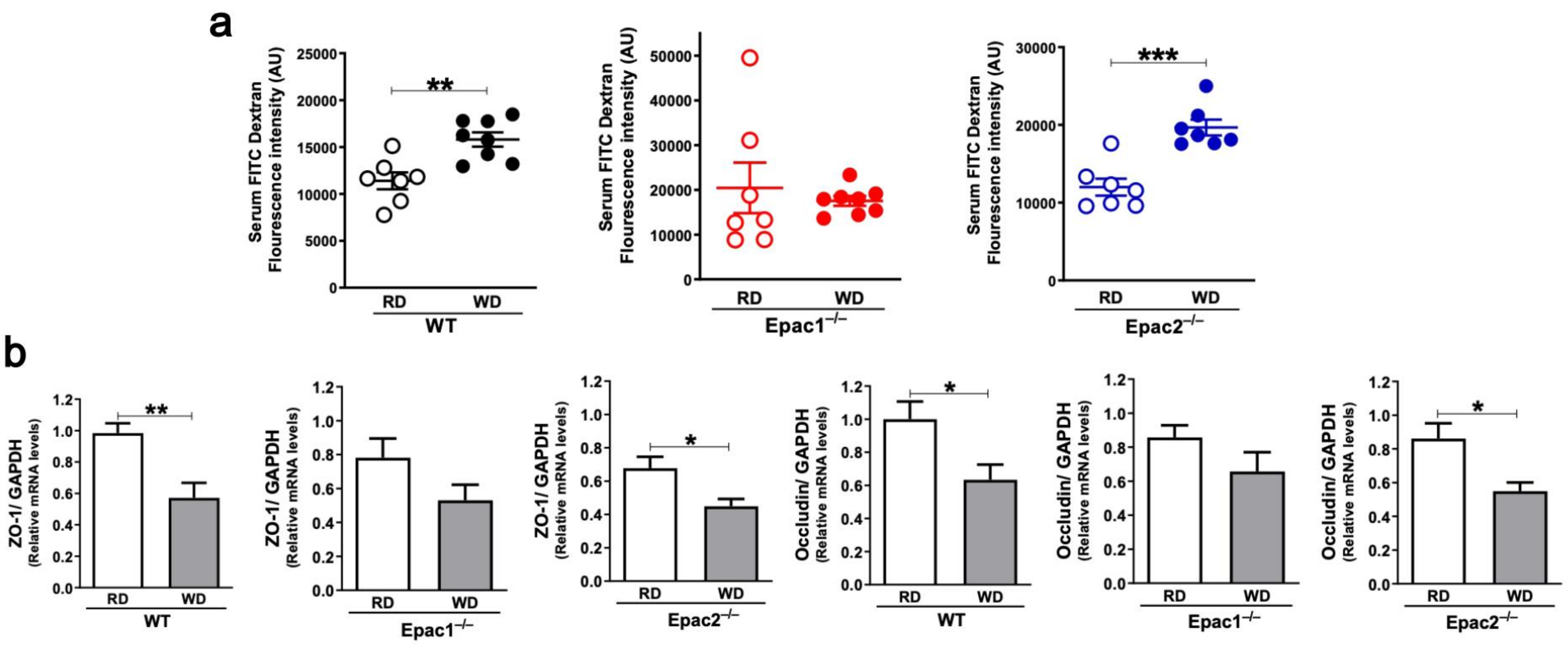

Fig S8. Gut permeability alterations in WT, Epac1$^{-/-}$ and Epac2$^{-/-}$ mice upon WD feeding compared to their respective RD-fed counterparts . The following data are from 16-week-old mice (8-week-old + RD or WD for 8 weeks). (**a**) *In vivo* gut permeability test (n=7-8/genotype/diet). (**b**) Relative mRNA expression of ZO-1 and Occludin in the jejunum determined by RT-qPCR (n=4-6/genotype/diet). Statistical significance *p<0.05, **p<0.01, ***p<0.01 was assessed by unpaired student's t test. All data are presented as mean ± SEM.

Fig. S9

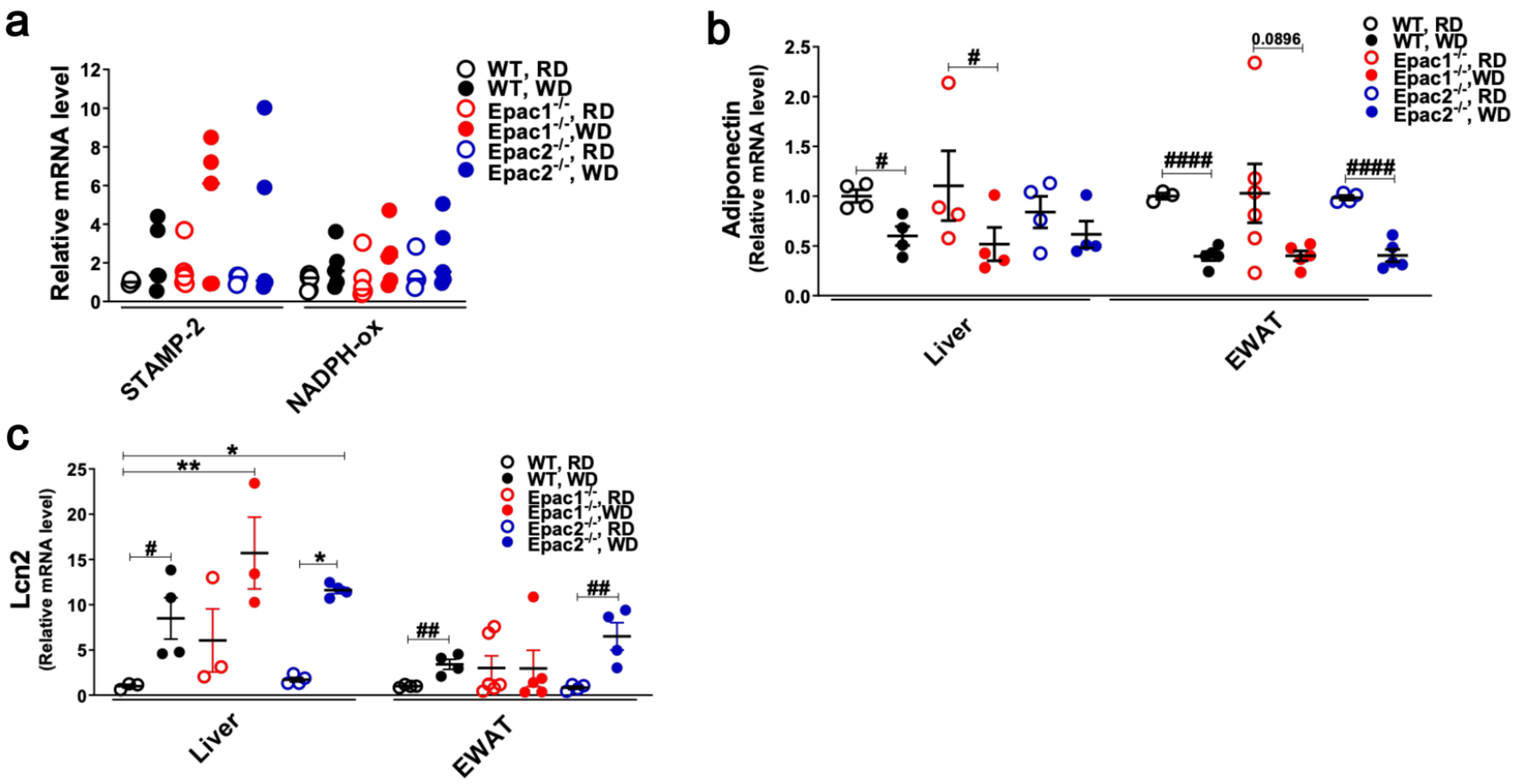

Fig. S9. Alterations in the mRNA levels of oxidative stress and adipokines in the liver and EWAT of WT, Epac1$^{-/-}$ and Epac2$^{-/-}$ mice upon WD feeding. All data are from 16-week-old mice (8-week-old + RD or WD for 8 weeks). Relative mRNA expression of (**a**) oxidative stress markers, STAMP-2, NADPH-ox in the EWAT; (**b**) Adiponectin in liver and EWAT and (**c**) Lcn2 in liver and EWAT. RPL19 was used as the internal control in the EWAT and GAPDH in the liver (n=3-6/group). Statistical significance *P<0.05 and **P<0.01 was assessed by two-way ANOVA with Tukey's test, and #P<0.05, #P<0.01 and ####P<0.0001 was calculated using unpaired student's t test. All data are presented as mean ± SEM.
